# Overnutrition is a risk factor for iron, but not for zinc or vitamin A deficiency in children and young people: a systematic review and meta-analysis

**DOI:** 10.1136/bmjgh-2024-015135

**Published:** 2024-04-09

**Authors:** Xiaomian Tan, Pui Yee Tan, Yun Yun Gong, J Bernadette Moore

**Affiliations:** School of Food Science and Nutrition, University of Leeds, Leeds, West Yorkshire, UK

**Keywords:** Child health, Nutrition, Public Health, Systematic review

## Abstract

**Introduction:**

Traditionally associated with undernutrition, increasing evidence suggests micronutrient deficiencies can coexist with overnutrition. Therefore, this work aimed to systematically review the associations between iron, zinc and vitamin A (VA) status and weight status (both underweight and overweight) in children and young people.

**Methods:**

Ovid Medline, Ovid Embase, Scopus and Cochrane databases were systematically searched for observational studies assessing micronutrient status (blood, serum or plasma levels of iron, zinc or VA biomarkers) and weight status (body mass index or other anthropometric measurement) in humans under 25 years of any ethnicity and gender. Risk of bias assessment was conducted using the American Dietetic Association Quality Criteria Checklist. Where possible, random effects restricted maximum likelihood meta-analyses were performed.

**Results:**

After screening, 83 observational studies involving 190 443 participants from 44 countries were identified, with many studies having reported on more than one micronutrient and/or weight status indicator. Iron was the most investigated micronutrient, with 46, 28 and 27 studies reporting data for iron, zinc and VA status, respectively. Synthesising 16 records of OR from seven eligible studies, overnutrition (overweight and obesity) increased odds of iron deficiency (ID) (OR (95% CI): 1.51 (1.20 to 1.82), p<0.0001, I^2^=40.7%). Odds appeared to be higher for children living with obesity (1.88 (1.33 to 2.43), p<0.0001, I^2^=20.6%) in comparison to those with overweight (1.31 (0.98 to 1.64), p<0.0001, I^2^=40.5%), although between group differences were not significant (p=0.08).

**Conclusions:**

Overnutrition is associated with increased risk of ID, but not zinc or VA deficiencies, with an inverted U-shaped relationship observed between iron status and bodyweight. Our results highlight significant heterogeneity in the reporting of micronutrient biomarkers and how deficiencies were defined. Inflammation status was rarely adequately accounted for, and the burden of ID may well be under-recognised, particularly in children and young people living with overnutrition.

**PROSPERO registration number:**

CRD42020221523.

**WHAT IS ALREADY KNOWN ON THIS TOPIC**

- Low-income and middle-income countries are increasingly facing a double burden of malnutrition, that is, the coexistence of undernutrition (stunting, wasting, underweight) with overnutrition (overweight and obesity).
- While the relationship between undernutrition and critical micronutrients for childhood growth and development (eg, iron, zinc and vitamin A (VA)) is well established, less is known about the risk of micronutrient deficiencies (MNDs) in children and adolescents with overweight or obese, a hidden form of malnutrition.
- There are limited data summarising associations between biomarkers of the most commonly limiting micronutrients and body weight status, particularly in children and young people.

**WHAT THIS STUDY ADDS**

- Overnutrition increases the risk of iron deficiency (ID), but not zinc or VA deficiencies.
- There is an inverted U-shaped relationship observed between iron status and bodyweight in children and young people, with IDs observed more frequently in both undernutrition and overnutrition.
- Studies conducted to date have been heterogeneous in terms of populations studied, diagnostic criteria and approaches to data analysis; few studies followed current guidelines for measuring inflammation and defining MNDs.

**HOW THIS STUDY MIGHT AFFECT RESEARCH, PRACTICE OR POLICY**

- Healthcare practitioners will increasingly recognise that children and young people living with overweight or obesity are likely to be iron deficient, resulting in improved clinical practice and care.
- More research needs to be conducted to examine MNDs and the double burden of malnutrition from currently under-represented countries.
- In future research investigating MNDs, there is a critical need for enhanced reporting and higher-quality evidence.

## Introduction

Deficiencies in micronutrients contribute to impaired immune function, poor growth and physical development and increased morbidity and mortality in children.^1 2^ Public health prevention strategies such as supplementation, fortification and nutrition education are therefore strongly encouraged by the WHO and UNICEF in low-income and middle-income countries (LMICs).^3 4^ Although great strides have been made in the last century to address micronutrient deficiencies (MNDs) and reduce childhood mortality rates, disappointingly in 2013 (median year of data collection), the global prevalence of deficiency in one or more micronutrients was estimated to be 56% in children under 5 years, although rates vary across countries.^5 6^

Among the micronutrients, deficiencies in iron, zinc and vitamin A (VA) remain particularly prevalent and causally associated with adverse health outcomes for children. Iron deficiency (ID) and ID anaemia (IDA) are major global health challenges affecting more than 1.2 billion people worldwide.^7^ In addition to ID, it is estimated that more than 25% of South Asia and sub-Saharan African populations are at risk of insufficient dietary zinc intake, with some countries particularly deficient.^8^ In a large population survey in Ethiopia, for example, the prevalence of zinc deficiency among young children was shockingly found to be 89%, improving somewhat in school children to 71%.^9^ Similarly, in Southeast Asia in Vietnam, in 2010 the prevalence of zinc deficiency was remarkably high (68%) in young children, improving to 42% in children over 5 years of age.^10^ Moreover, although large-scale VA supplementation programmes have been implemented in many countries,^2^ subclinical VA insufficiency is still prevalent in Vietnam (10.1%, young children aged 5–75 months, data from Vietnam nationwide food consumption survey^10^), and Africa (Ethiopia, 45.4% for 6–72-month-old children, from meta-analysis in 2020^11^), and often observed in the context of multiple MNDs.^12^

Historically, MNDs were considered as one of the four forms of undernutrition, alongside wasting, stunting and underweight,^13^ and a particular concern for LMICs where undernutrition may be the leading cause of childhood mortality for children under 5.^2 14^ However, increasingly, it is recognised that MNDs also occur in the context of overweight and obesity.^15^ Deficiencies in iron,^16^ zinc^17^ and VA^18^ have been observed in adults living with overweight and obesity and associated metabolic diseases. Typically associated with nutrient-poor, energy-dense diets, the presence of multiple MNDs in the absence of an energy-deficit diet has been described as ‘hidden hunger’.^19^ While in high-income countries obesity is associated with ultra-processed foods that are high in fat, sugar, salt and energy, in LMICs overweight and obesity are often associated with poverty and monotonous diets with limited choices of low-cost energy-dense staples such as corn, wheat, rice and potatoes.^19 20^ Even in high-income countries, childhood obesity is strongly linked to poverty and socioeconomic and health disparities.^21^

Moreover, many LMICs are now facing a double burden of malnutrition with the coexistence of an increasing prevalence of overnutrition alongside undernutrition.^22^ This is intricately linked to the rapid increase in the global prevalence of obesity, especially in children aged 5–19 years in recent decades.^23^ Deficiencies in multiple micronutrients including iron,^24 25^ zinc^17 26^ and VA^27 28^ have also been observed in children living with malnutrition. Indeed, the term ‘triple burden of malnutrition’ aims to underscore the coexistence of MNDs alongside undernutrition (stunting, wasting, underweight) and overnutrition.^29^

While increasing evidence now suggests MNDs are associated with overnutrition as well as undernutrition, to date there have been only a limited number of reports summarising associations between biomarkers of micronutrient status and obesity in children and young people. In one meta-analysis of multiple comorbidities associated with obesity in children, higher OR for both vitamin D deficiency (OR (95% CI): 1.9 (1.4 to 2.5)) and ID (OR 2.1, 95% CI 1.4 to 3.2) were found in children under age 10 living with obesity.^30^ Similarly, a separate meta-analysis of vitamin D deficiency in children and adolescents aged 0–18 years also found a positive association between obesity and vitamin D deficiency (OR 1.4, 95% CI 1.3 to 1.6).^31^ However, to our knowledge the risks of iron, zinc and VA deficiencies, the most frequently limiting micronutrients in children and young people, have not been collectively examined. Therefore, in this work, we aimed to systematically review the associations between iron, zinc and VA status and body weight in children and young people, using meta-analyses to summarise where sufficient data existed.

## Methods

This review was conducted following the Preferred Reporting Items for Systematic Reviews and Meta-Analyses (PRISMA) guidelines^32^ and was registered at PROSPERO (CRD42020221523).

### Search strategy

The Ovid Medline, Ovid Embase, Scopus and Cochrane databases were systematically searched through 19 April 2023. A combination of keywords and related medical subject headings were used from three main themes: (1) population: infants, children, adolescents and young adults; (2) malnutrition indicators, including undernutrition and overnutrition; and (3) blood micronutrient indicators for iron, zinc and VA. The specific search strategies developed for each database are reported in online supplemental tables 1–4.

10.1136/bmjgh-2024-015135.supp1 Supplementary data

### Inclusion and exclusion criteria

Observational studies in humans under 25 years of age,^33^ of any nationality, gender or ethnicity, with undernutrition or overnutrition diagnosed by body mass index (BMI) or other anthropometric measurement, were included. In addition to be included, studies had to have reported as primary outcomes either: a blood or serum test of micronutrient level, the OR or relative risk of MNDs, or the linear regression between a micronutrient indicator and weight status. Records excluded from this review were: non-English articles, short reports, communications, address, case reports, comments, letters, editorial matters, meta-analyses, news, reviews, conference abstracts, studies with unrepresentative samples (either convenience sampling or n<100), studies not reporting primary outcomes of interest, studies reporting associations with combined MNDs and studies of micronutrient supplement interventions. Studies that assessed anaemia diagnosed with only haemoglobin (Hb) were excluded, as not specific to ID.

### Study selection

The screening of identified studies was managed using the web-based Rayyan software.^34^ The selection of the eligible studies for inclusion in this review was assessed and agreed by two independent reviewers (XT and PYT). Disagreement between reviewers was resolved by discussion or by third reviewer if necessary.

### Data extraction

A standardised data extraction form was used to extract the following information: first author, year of publication, year of study, study design, country, participants' characteristics (eg, sample size, recruitment, gender, age, socioeconomic status, etc), exposure (eg, anthropometric indicators and blood/serum levels of micronutrients of interest), control or non-exposure (eg, children who were not malnourished), outcome measures (eg, changes in anthropometric indicators in the presence of MNDs or otherwise changes in the blood micronutrients levels in different anthropometry groups), main findings (eg, differences in mean, β coefficient, OR, relative risk) and funding or sponsorship.

### Risk of bias assessment

Risk of bias was assessed by two reviewers using the Quality Criteria Checklist developed by the Academy of Nutrition and Dietetics.^35^ Any disagreement was resolved by discussion and by a third reviewer if necessary. This tool has 10 validity questions: (1) clear research question, (2) non-biased participant recruitment, (3) group comparability, (4) report of withdrawals/participant ratio, (5) blinding, (6) study procedures description, (7) outcome, (8) appropriate statistical method and adequate adjustments, (9) conclusion supported by results and (10) conflict of interest. The overall rating was defined as either positive (majority of criteria above were met, in which criteria 2, 3, 6 and 7 must be met), neutral (any one criterion of criteria 2, 3, 6 and 7 was not met) or negative (six or more of the criteria not being met).

### Data analysis

The associations (eg, OR, relative risk, linear regression) between the blood micronutrient levels of interests and anthropometry status were curated in tables according to micronutrient. If no association data was provided, the comparison of mean micronutrient levels between different anthropometry groups was included in the table. For the convenience of summarising, the main findings of each study were concluded as either direct associations (micronutrient levels increased with increasing weight status indicators) or inverse associations (decreased micronutrient levels—increasing risk of MNDs—with increasing weight status indicators).

Weight status indicators included BMI-for-age z score (BAZ) and BMI used to categorise overnutrition. While for undernutrition, height-for-age z score, weight-for-age z score and weight-for-height z score were used to categorise stunting, wasting and underweight, respectively, following the WHO growth chart, obesity work taskforce or national standard in respect to each country. Micronutrient status indicators included blood micronutrient levels (iron, zinc, retinol), Hb, iron profile, β-carotene, retinol binding protein or diagnosis of ID, IDA, zinc deficiency or vitamin D deficiency (VAD). The diagnostic criteria of MNDs mostly followed either WHO criteria or the International Zinc Nutrition Consultative Group standard or country-specific standards for defining MNDs.

Where there were at least five studies^36^ assessing the risk of MNDs in malnutrition groups comparing with normal weight group, random effects restricted maximum likelihood meta-analyses were conducted to estimate the OR with 95% CI using Stata V.18 (Stata Corporation, College Station, Texas, USA). Heterogeneity across the studies was evaluated using I^2^. Sensitivity analysis was conducted by using the leave-one-out method, to compare the pooled OR before and after eliminating each study at a time. To detect publication bias, the asymmetry of the funnel plot was examined. Statistical significance was set at p<0.05. To avoid overestimation of power, the gender-stratified data were not included in meta-analysis if overall population data were reported by the study. All graphics were produced in either Stata or using the R-package ggplot2,^37^ ggalluvial^38^ and rworldmap^39^ in the R environment.^40^

#### Patient and public involvement

It was not appropriate to involve patients or the public in the design or conduct of our research. However, members of the general public, including children and young people, were consulted about dissemination materials.

## Results

**Figure 1:**
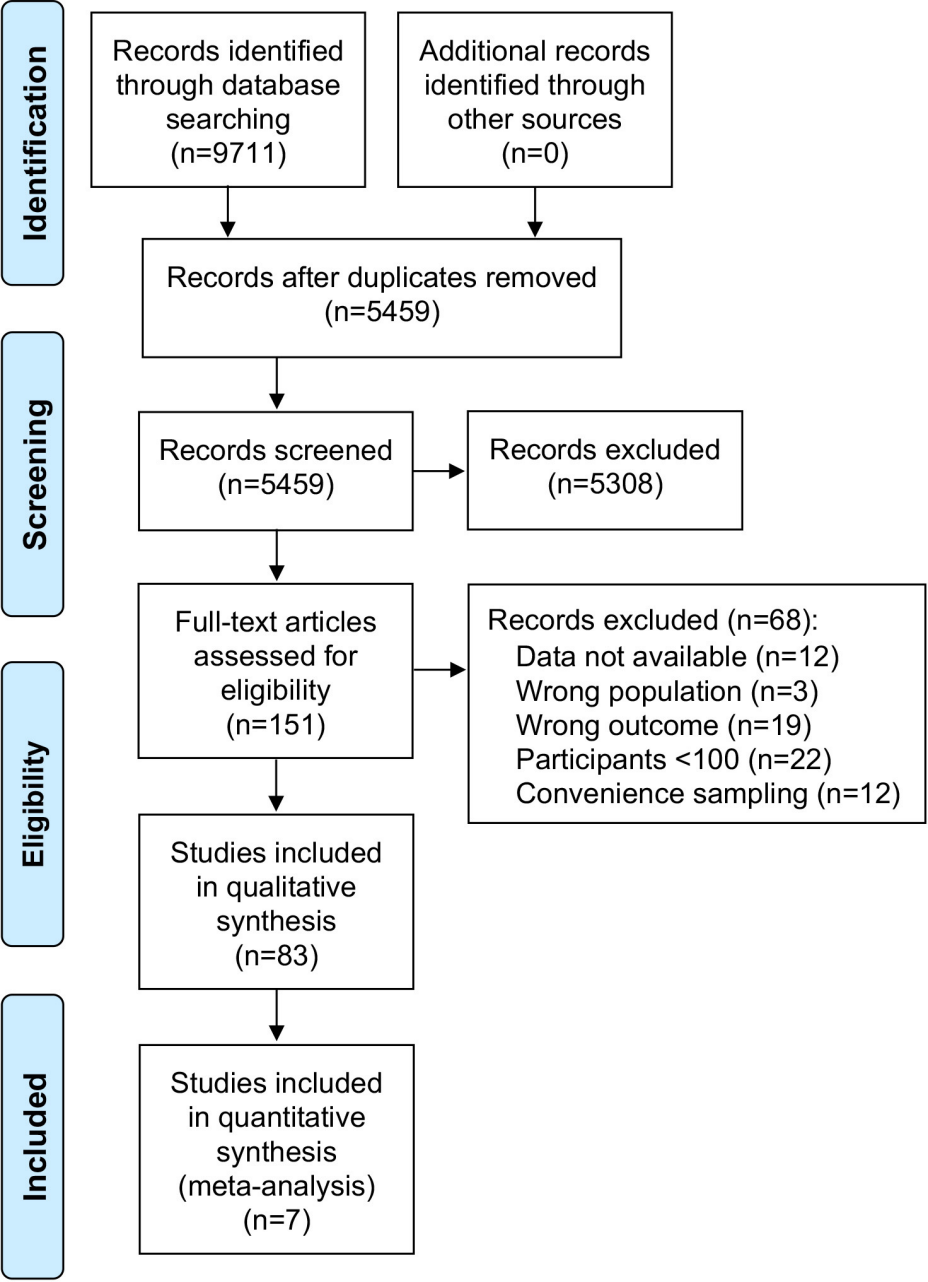
Flow diagram of the systematic identification and selection of articles.

Using systematic search strategies, a total of 9711 articles were initially identified from four databases, of which 5459 articles remained after deduplication. After title and abstract screening, the full texts of 151 articles were assessed to check for eligibility. From these, 83 observational studies met the inclusion criteria and 7 studies were eligible for meta-analysis (figure 1).

**Figure 2:**
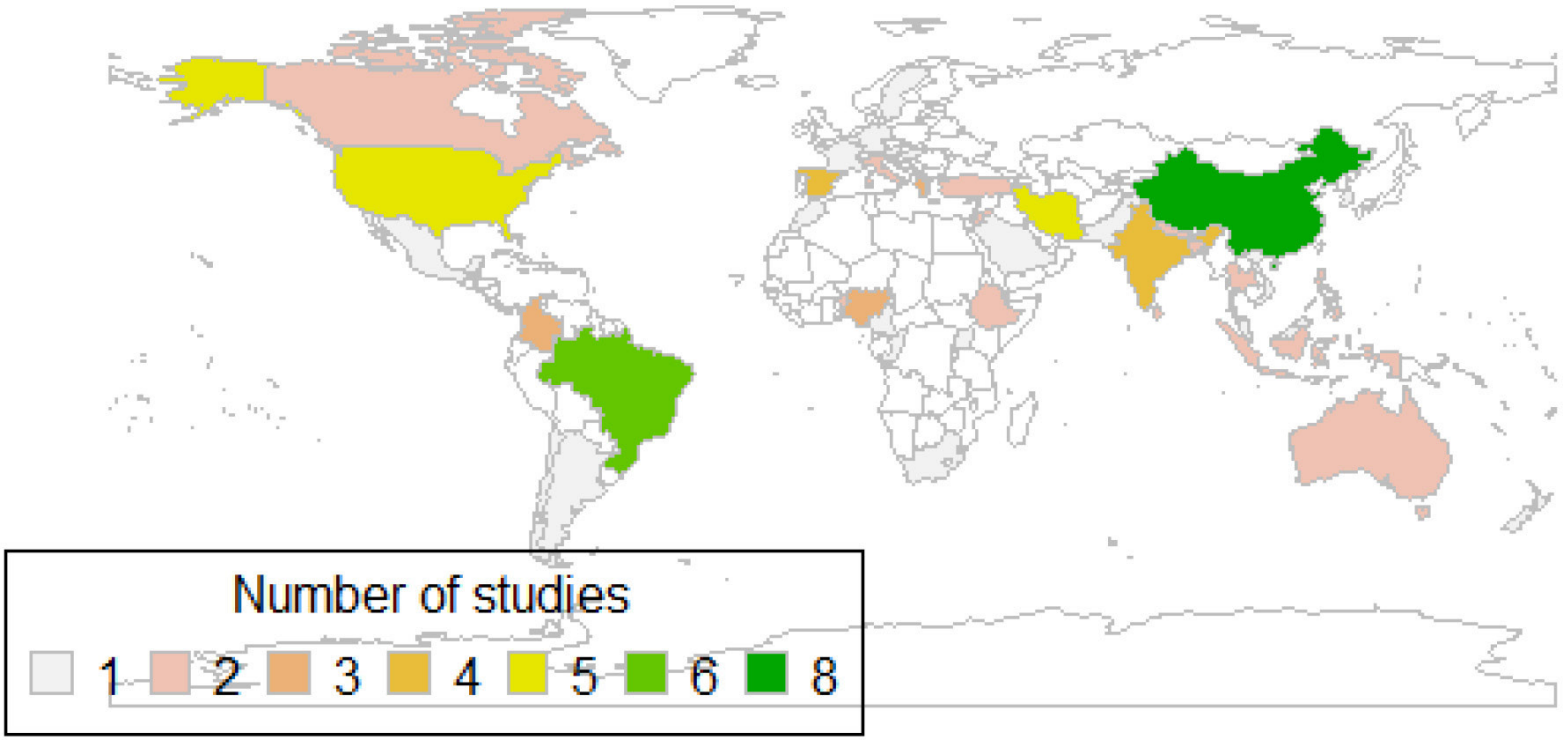
World map showing the populations represented in the included articles.

Overall, the 83 studies involved a total of 190 443 participants from 44 countries (figure 2). The majority (n=74, 89.1%) were cross-sectional studies, and the number of participants in individual studies ranged from n=101^41^ to n=64 850 (the China National Nutrition and Health Survey of Children and Lactating Mothers study, 2016–2017^42^), with a median n=675 participants. Notably, many studies assessed more than one micronutrient and/or weight status indicator at the same time. Iron was the most investigated micronutrient, with a total of 46 (n=10, 21 and 15 for both overnutrition and undernutrition, respectively), 28 (n=4, 8 and 16 for both overnutrition and undernutrition, respectively) and 27 (n=5, 12, 10 for both overnutrition and undernutrition, respectively) studies identified for iron (online supplemental table 5), zinc (online supplemental table 6) and VA (online supplemental table 7), respectively.

### Associations between iron and weight status

The characteristics and findings of the studies that reported associations between iron status and weight status in children and young people (n=46) are summarised in online supplemental table 5. 10 studies assessed both sides of malnutrition, whereas 21 studies focused on overnutrition and 15 studies focused on undernutrition. A broad range of biomarkers for ID were used by the different studies, and most studies reported more than one (online supplemental table 5). In 2020, the WHO revised their guidance on the use of ferritin to assess iron status in individuals and populations, providing thresholds of <15 µg/L for healthy individuals above 5 years of age and <70 µg/L for individuals with infection or inflammation (<12 µg/L or <30 µg/L are used for children under 5). While current WHO guidance is that ferritin, both an iron storage protein and an acute phase reactant associated with inflammation,^43^ should not be used alone to diagnose ID without other iron profile biomarkers or corrections for inflammation,^44^ a minority of studies did rely on ferritin alone.^25 45–48^ Likewise, serum iron alone should not be used in isolation to diagnose ID as serum iron levels will be reduced in the context of infection or inflammation,^49^ but was used in a handful of studies.^43 50 51^ In risk of bias assessment, most studies were found to be of positive quality (28 out of 46; online supplemental table 5). Only three studies were found to be of negative quality.^50–52^ Overall, the most common reasons for studies being rated either neutral or negative quality were either the lack of: (1) an appropriate statistical approach, such as multivariate logistic or linear regression with adequate adjustments, eg, inflammation status or menarche, or (2) essential information such as group comparability, information of withdrawals or limitation of the study (online supplemental table 8).

**Figure 3:**
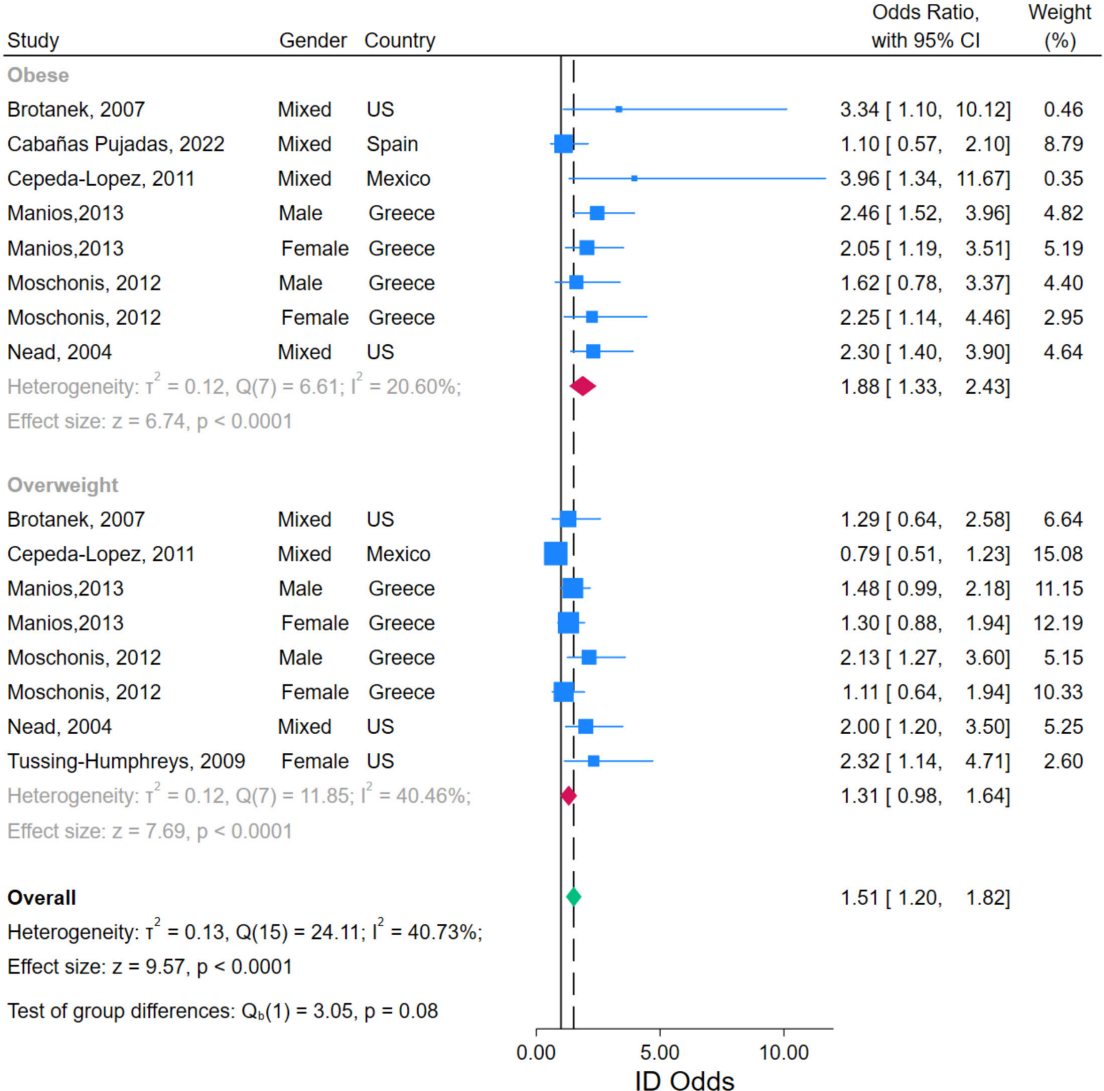
Forest plot of reported associations between overnutrition and odds of iron deficiency (ID).

A total of 16 records reported OR from seven eligible studies enabling our calculation of the pooled OR for ID with overnutrition (overweight or obesity) using the normal weight groups as reference. These studies assessed overnutrition and ID in North American (USA^45 53 54^ and Mexico^55^) and European (Spain^56^ and Greece^57 58^) populations, and, in risk of bias assessment, all were assessed as positive (good) quality. The random-effects model meta-analysis indicated a pooled OR (95% CI) of 1.51 (1.20 to 1.82), p<0.0001, for ID with overnutrition (figure 3). Heterogeneity was moderate with an I^2^ value of 40.73%, likely a result of the diversity of ethnicities, ages and genders studied as well as the broad range of biomarkers for ID and overnutrition used by the different studies. Subgroup analyses suggested increased risk of ID in those living with obesity OR (95% CI): 1.88 (1.33 to 2.43), p<0.0001, compared with overweight OR (95% CI): 1.31 (0.98 to 1.64), p<0.0001; however between group differences were not statistically different (p=0.08), likely driven by greater heterogeneity in the overweight versus obesity data (I^2^: 40.46% vs 20.0%, figure 3).

Similarly, no differences were observed between studies that reported data separately for males and females versus those that reported data from mixed-gender populations (p=0.47, online supplemental figure 1). Funnel plot analyses suggested some asymmetry driven by two outliers (online supplemental figure 2). These studies reported higher prevalence of ID in both overweight and obese mixed-gender populations, with much larger SEs observed in the (fewer) children with obesity (figure 3).^53 55^ As they contributed only small weights to the meta-analysis (Brotanek *et al*: 0.46%, Cepeda-Lopez *et al*: 0.35%), this is a small-study effect rather than non-reporting bias, and indeed funnel analyses with fewer than 10 studies should be interpreted cautiously.^59^ Sensitivity analyses indicated robustness in the overall results as the effect size was not significantly changed after removing any of the studies (online supplemental figure 3).

**Figure 4:**
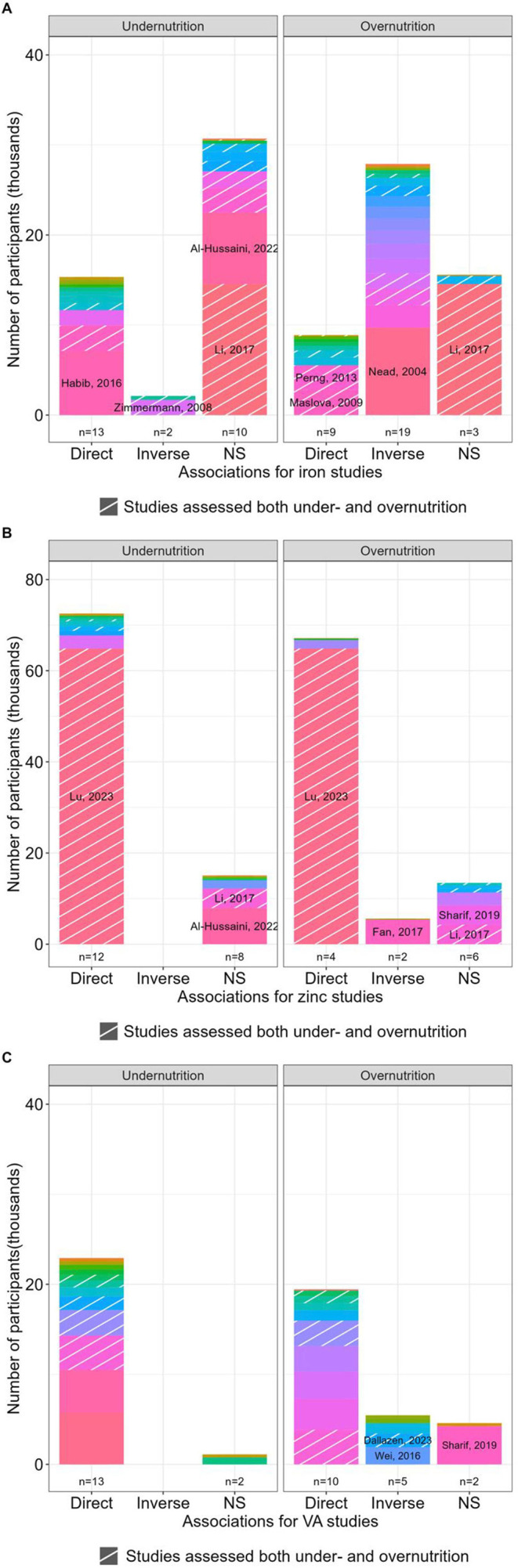
Summary stacked bar plots showing participant numbers, grouped by the direction of associations, in studies examining overnutrition or undernutrition: (A) iron, (B) zinc and (C) VA. Studies where participants represented >25% of the total population in each category were labelled. NS: not significant VA: vitamin A.

Given that the meta-analysis incorporated only a small subset (7 of 46) of iron studies, to synthesise the findings across all studies, we examined the numbers of participants in the reports concluding either direct, inverse or no significant associations between ID and nutrition status (figure 4A). These data illustrate surprising heterogeneity in the conclusions of individual studies and show where individual studies contributed disproportionally larger populations. In the case of iron and undernutrition, while 13 reports that included 15 335 participants found a direct association (ie, better iron status found with normal bodyweight in comparison to undernutrition), surprisingly 10 studies that included 30 705 participants found no association and 2 concluded an inverse association (figure 4A). Notably, the two studies that found an inverse association had reported linear regression between iron and both undernutrition and overnutrition (BMI 14–32^60^ and BAZ between −4 and 2^57^). However, the associated scatter plots suggest piecewise regression would be more appropriate as very low ferritin values were observed in both the lowest and highest quintiles of nutrition status. While the data for overnutrition appear clearer, with 19 reports including 27 894 participants concluding an inverse relationship (ie, worsening iron status as weight status moves from normal to overweight and obesity), nonetheless 9 reports with 8884 participants concluded a direct association and 3 found no association (figure 4A).

While these conflicting results likely reflect different populations studied and the challenges of ID diagnosis, nonetheless, we conclude the data collectively suggest an inverted U-shaped relationship between iron status and bodyweight, with children and young people living with overnutrition having a higher risk (pooled OR (95% CI): 1.51 (1.20 to 1.82)) of ID alongside those with undernutrition.

### Associations between zinc and weight status

The 28 studies that examined zinc status and either undernutrition or overnutrition were similarly diverse in their approaches and findings (online supplemental table 6). In particular, how authors compared groups statistically (eg, mean, median, quartile (Q): Q2–4 vs Q1) varied between studies and precluded any meta-analysis. Although the majority of studies (27 out of 28) measured either plasma or serum zinc, 1 study used whole blood and concluded a direct association,^61^ however, measurements of zinc in whole blood may overestimate zinc levels as whole blood zinc level was five-fold higher than plasma due to red blood cells.^62^ We note only two studies^26 63^ used international consensus-based thresholds (57–74 µg/dL depending on age group, sex, time of day and time since last meal^64^) for defining deficiency, which may explain some of the heterogeneity in conclusions between studies here. In addition, although plasma or serum zinc concentration is the most used biomarker of zinc status, there are well-described issues with sensitivity and specificity, as well as challenges with sample collection and storage, that render the assessment of dietary zinc status in humans challenging.^65^ In the risk of bias assessment, most studies (17 out of 28) received a positive quality rating.

Although a direct relationship between zinc status and undernutrition was concluded by the majority of studies (12 of 20 accounting for 72 537 participants; figure 4B), the results from reports on overnutrition were more contradictory. While four studies concluded direct associations between zinc levels and BMI,^42 61 66 67^ two found inverse relationships^68 69^ and six studies, accounting for 13 440 participants in four countries (three from Iran,^70–72^ and one from Colombia,^73^ Australia^74^ and China^75^), concluded no association between zinc status and overweight (figure 4B). Among the studies reporting direct associations with both undernutrition and overnutrition was an exceptionally large population study from China with >64 000 participants.^42^ While in this cohort stunting conferred a higher risk of zinc deficiency (OR (95% CI): 1.44 (1.19 to 1.75)), and overweight and obesity appeared to confer protection (overweight: 0.88 (0.84 to 0.96); obesity: 0.78 (0.70 to 0.86)), serum zinc values were not hugely different between groups and within adequate population ranges,^64 76^ with 86.0 µg/dL (77.0 to 96.0) reported for children with stunting and 91.0 µg/dL (82.0 to 101.0) for children living with obesity.^42^ Relatedly, in a large American cohort (n=5404 children aged 5–19 years) the overall population mean (range) of zinc levels was similarly adequate, 82.7 µg/dL (38.9 to 198.6).^68^

In sum, while the data were more limited for zinc, they suggest an exponential plateau curve relationship between zinc status and bodyweight, with children and young people living with severe undernutrition having the highest risk for zinc deficiencies, and no evidence for a higher risk for zinc deficiency with overnutrition.

### Associations between VA and weight status

The studies that investigated VA status in relation to body weight were similarly heterogenous in their approaches to assessing both VA and nutrition status (online supplemental table 7). VA was commonly assessed by measuring plasma/serum retinol levels, although some studies measured either serum carotenoids, b-carotene or retinal binding protein levels. However, plasma/serum retinol concentration is under tight homeostatic control and does not reflect VA status until body stores are extremely low or very high.^77^ Where low serum retinol was defined, WHO cut-offs (retinol<0.7 µmol/L, equivalent to 20 µg/dL^78^) were most often but not always used. These values are not sensitive to moderate VA deficiencies, and, similarly, with zinc, studies often examined results by comparing bottom and top quartiles of the biomarkers measured. As with the zinc studies, variability in the outcomes measured and statistical approaches between studies prevented us from performing meta-analysis, and the majority of studies received positive quality ratings (17 out of 27; online supplemental table 7).

While a direct relationship between undernutrition and VA deficiency was clear with 13 of 15 studies concluding a direct association, the other 2 reported no association (figure 4C), the data for associations between overnutrition and VA deficiency were more contradictory. Although in favour of a direct relationship between VA status (n=10 of 17 studies, accounting for 19 443 participants), 5 found an inverse association and 2 found no association (figure 4C). In the 10 studies that found a direct relationship, these were reported in a variety of ways, such as, lower risk of VAD in the context of overweight or obese^75 79–83^ or elevated serum levels of VA or plasma retinol in children with overweight or obesity.^73 84–86^ Interestingly, of the five studies that concluded an inverse association between overnutrition and VA deficiency, four focused on Brazilian children. The associations were reported either as increased risk of VAD^28^ or risk of low carotenoids,^87 88^ for overweight children aged 5–19 years with around 500 participants in each study. While in one case, low plasma retinol (<0·70 µmol/L) was associated with overweight in Brazilian children aged 12–59 months (n=1503).^89^ The fifth, non-Brazilian, study assessed 7–11 years old children (n=1928) in Chongqing, China,^90^ and found that obesity increased the risk of VAD, OR (95% CI): 2.37 (1.59 to 3.55).

In the context of the tight homeostatic mechanisms for plasma retinol levels,^77^ we conclude that similar to zinc, the data suggest an exponential plateau curve relationship between VA status and bodyweight, with children and young people living with severe undernutrition having the highest risk for VA deficiency and no evidence for a higher risk for VA deficiency with overnutrition.

**Figure 5:**
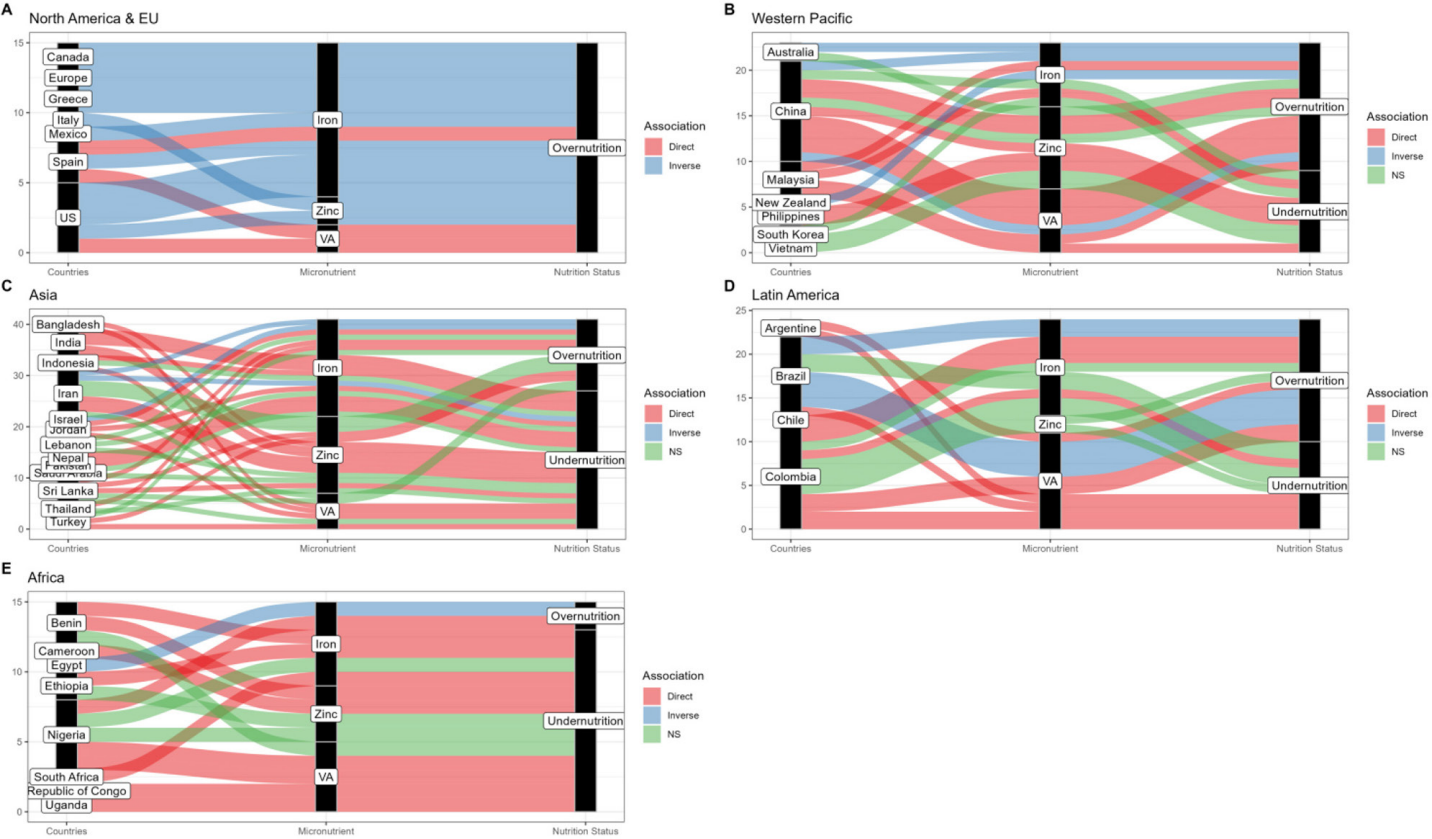
Sankey plots illustrating regional differences in study characteristics and conclusions: (A) North America and Europe, (B) Western Pacific, (C) Asia (excluding Western Pacific), (D) Latin America and (E) Africa. Each line represents a reported association for different micronutrient and nutritional status in different country. The left black column illustrates the countries studies originated from, the middle black column illustrates the relative proportion of studies investigating either iron, zinc or vitamin A (VA) and the right black column illustrates the relative proportion of studies investigating either overnutrition or undernutrition. NS: not significant.

Lastly, regional differences were observed both in terms of which micronutrients were more frequently investigated and whether populations with undernutrition or overnutrition or both were investigated (figure 5). Studies from North America and Europe (figure 5A) focused entirely on overnutrition and largely on iron, whereas reports from the Western Pacific (figure 5B), Asia (figure 5C) and Latin America (figure 5D) assessed both undernutrition and overnutrition and most studies in Africa (figure 5E) focused on undernutrition.

## Discussion

To our knowledge, this is the first systematic review of the associations between weight status in children and young people and iron, zinc and VA deficiencies, to concurrently examine associations with both undernutrition and overweight, that is, the double burden of malnutrition. Notably, our results suggest an inverted U-shaped relationship between iron status and bodyweight, with both undernutrition and overnutrition increasing risk for ID. In meta-analyses, children and young people living with overnutrition had a higher risk (pooled OR (95% CI): 1.51 (1.20 to 1.82)) of ID, with those living with obesity having even higher risk (pooled OR (95% CI): 1.88 (1.33 to 2.43)) compared with those with normal weight. In contrast, zinc and VA deficiencies were most observed in children and young people with undernutrition. While in aggregate, the studies underscore the increased risks for iron, zinc and VA deficiencies with undernutrition in childhood and adolescence, there was more heterogeneity in study conclusions (ie, several studies finding no relationship) for iron and zinc. Such heterogeneity in part relates to the methodological challenges of assessing the status of these micronutrients in humans. Indeed, our results highlight how variable the approaches to measuring and reporting dietary nutrient deficiencies have been, as well as regional data gaps, with important implications for future study design.

Excess adiposity is associated with low-grade inflammation, widely proposed to be the mechanism behind reduced iron status observed in adults living with obesity.^91 92^ With the expansion of adipose tissue comes macrophage infiltration and release of inflammatory cytokines such as interleukin-6 (IL-6), which stimulate the synthesis of acute phase proteins such as ferritin and hepcidin, the main regulator of systemic iron homoeostasis.^91^ Hepcidin prevents iron absorption from the enterocytes and iron release from splenic macrophages, thereby reducing circulating iron levels overall.^92^ In acute infection, this serves to deprive a pathogen of iron,^93^ but in the context of chronic disease with prolonged immune activation can lead to anaemia of chronic disease.^94^ The results of our meta-analysis, showing a higher risk of ID with overnutrition, particularly in children and young people living with obesity, are consistent with those seen in adults^16^ and suggest inflammation-mediated increases in hepcidin as the potential mechanism. This would explain the difference between the observation of ID, but not zinc or VA deficiencies, in children with overweight or obesity. Examining hepcidin and inflammatory markers before and after weight loss intervention would permit testing this association as a causal mechanism. However, as noted in a recent review of studies investigating hepcidin and IL-6 levels in children with obesity,^95^ this has only rarely been done. But in two small studies, one 8-month exercise intervention (n=73)^96^ and one 6-month weight loss intervention (n=15),^97^ decreases in IL-6 and hepcidin were observed in conjunction with weight loss and improvements in iron status, in line with this hypothesis.

Inflammatory status is particularly relevant as ferritin, the primary blood biomarker of iron status used, was consistently found higher in obese groups compared with children with normal weight, with direct linear association reported in multiple studies.^25 46–49 56 66 85 98–105^ However, as ferritin is an acute phase reactant, inflammation can obscure ID^106^. Therefore when the WHO reviewed their guidance on the use of ferritin to assess iron status in 2020,^44^ they made the strong recommendation that in areas of widespread infection or inflammation, serum ferritin should be measured alongside both C-reactive protein (CRP) and α-1 acid glycoprotein (AGP), which reflect different phases of the immune response from acute infection to chronic inflammation. In addition, they recommend that thresholds to define ID in individuals with infection or inflammation should be raised to 30 mg/L for children under 5 and 70 mg/L serum ferritin for all age groups over 5.^44^ However, very few studies (n=4 out of 23 studies that used ferritin with or without other biomarkers to define ID) measured and excluded participants with elevated CRP level.^46 104 105 107^ Only one study measured AGP^108^ and only two studies from Colombia^85^ and Brazil^109^ used the higher thresholds for ferritin to define ID in the context of inflammation. Interestingly, the WHO serum ferritin concentration thresholds were based on expert opinion,^44^ and a recent analysis of serial cross-sectional National Health and Nutrition Examination Survey data concluded thresholds for ferritin for iron-deficient erythropoiesis should be higher, at least for healthy American children and women.^110^ Specifically, from regression models of the distributions of Hb and soluble transferrin receptor with serum ferritin in 2569 and 7498 healthy children under 5 and women (15–49 years old), they derived 20 mg/L for children under 5 and 25 mg/L in women as physiologically based serum ferritin thresholds for ID. These data suggest that the global burden of ID in children and young people may well be underestimated.

In contrast to iron, our results show that zinc and VA deficiencies were most observed in undernutrition, with limited evidence for a higher risk for deficiency with overnutrition. While variability in the outcomes measured and statistical approaches between studies precluded meta-analyses, critical review of the data in aggregate suggests an exponential plateau curve relationships between zinc or VA status and bodyweight, with children and young people living with severe undernutrition having the highest risk for these MNDs. Nonetheless, considerable uncertainty in the evidence base remains, particularly for overnutrition. This is not least of all because of efficient homeostatic mechanisms that buffer plasma zinc and VA levels to either dietary deficiency or excess, thereby maintaining plasma concentrations within a narrow range and preventing toxicity in the case of VA.^77 111^ Therefore, more moderate deficiencies in overnutrition may be masked. In addition, mechanistically there are complicated interactions between the micronutrients themselves (eg, VA deficiency impairs iron mobilisation and VA supplementation will improve Hb concentrations^111^) and between overnutrition, inflammation and micronutrient metabolism.

Indeed, our comprehensive review highlights the challenges of micronutrient assessment at an individual and population level with important implications for future study design and reporting in the context of the double burden of malnutrition. The most commonly used biomarkers of micronutrient status (plasma/serum ferritin, zinc and retinol levels) are sensitive to a variety of stimuli, including infection and inflammation,^78^ and a primary recommendation for future work investigating MNDs in either undernutrition or overnutrition is that CRP and AGP are routinely measured and inflammation accounted for in line with current expert guidelines. While in the case of iron, ferritin increases with inflammation and ID may be underestimated and thresholds for deficiency should be raised^112^; in the cases of zinc and VA, deficiencies may be overestimated as both plasma/serum zinc and retinol are, at least transiently, lowered in acute infection or inflammation.^113 114^ Conversely, additional challenges with zinc measurement exist as careful sample collection and handling is critical to prevent haemolysis or contamination from adventitious zinc in the environment,^64^ which may mask deficiencies, a potential confounder in the studies that surprisingly did not find zinc deficiency associated with undernutrition. If CRP and AGP are measured, inflammation can be adjusted for by either exclusion, the use of correction factors or linear regression approaches recently proposed by the Biomarkers Reflecting Inflammation and Nutritional Determinants of Anaemia project.^115^ Regression approaches have now been systematically developed and used to adjust for ferritin,^116^ zinc^114^ and VA^113^ values on a continuous scale.

In addition, our work highlights that many studies did not apply consensus-based cut-offs for defining either iron, zinc or VA deficiencies but rather compared lower to upper quartiles of plasma/serum biomarkers within their populations, often not specifying the measured ranges of biomarkers within quartiles. Such calculations may overestimate risks of deficiencies in largely replete populations, and their widespread use may explain some of the seeming contradictory conclusions. Lastly, our data highlights regional data gaps and differences in study focus, with most studies from Africa and Asia focused on undernutrition and those from North America and Europe focused entirely on overnutrition. This is concerning as both Africa and Asia experienced dramatic increases in the number of overweight children under 5 between 2000 and 2017 (from 6.6 to 9.7 million children in Africa and from 13.9 to 17.5 million children in Asia).^117^ Moreover, the regions of Africa and Asia have the highest double burden of malnutrition with large numbers of stunted and wasted children and the number of stunted children under 5 having increased from 50.6 to 58.7 million children in Africa.^117^ These stark data underscore that the investigation of MNDs in relation to the double burden of malnutrition remains critically important for child health. Interestingly, a study published after our literature search assessed the risk of ID (serum ferritin <12 µg/L) in relation to BMI in healthy children (n=2575, aged 12–29 months) living in Canada.^118^ A rare example of a study investigating the double burden of malnutrition in a high-income country, it is notable that no association between underweight and ID was found. However, they did find that toddlers with overweight (BAZ>2) had higher risk of ID (OR=2.15 (1.22 to 3.78), p=0.008) in line with the results of our meta-analysis (we note that as they employed convenience sampling, the study did not meet our inclusion criteria).

This is the first study to concurrently, comprehensively review the associations between iron, zinc and VA deficiencies and the double burden of malnutrition in children and young people. In general, undernutrition groups were reported to have lower plasma/serum ferritin, zinc and retinol levels or found to have higher risks for iron, zinc and VA deficiencies. Notwithstanding evidence gaps, we conclude that overnutrition was associated with ID, but not zinc or VA deficiencies. Strengths of this work included our comprehensive search strategy with robust inclusion and exclusion criteria and a priori registration of detailed review protocol in line with PRISMA guidelines^32^ and using the Population-Investigation-Comparison-Outcome approach.^119^ In addition, this systematic review included populations across the globe (including North American and Europe, Latin American, Western Pacific, Asia and Africa continents), which allowed us to identify regional data gaps and differences in term of their nutritional outcomes or micronutrients of interests and the associations reported. While we excluded studies with small sample size (<100) and those that employed convenience sampling methods to reduce the bias, this review has also highlighted the importance of addressing the challenges and weaknesses in study design, analytical methods and diagnostic criteria for MNDs, to ensure the validity and reliability of data in future study for interpretation. Nonetheless, this work also has some limitations. First, all observational studies are limited by confounding and causality should not be inferred. Second, only four databases (Medline, Scopus, Embase and Cochrane) were searched, and only studies written in English were included, which may have limited the scope. Last but not least, MND status assessment is not trivial in humans, and the underlying studies included in this review were heterogeneous in the populations studied and approaches taken, conferring some limitations in data interpretation.

## Conclusion

Iron, zinc and VA deficiencies were commonly associated with undernutrition in children and young people. Overnutrition increased the risk of ID, but not zinc or VA deficiencies, with an inverted U-shaped relationship observed between iron status and bodyweight. Heterogeneity between studies was attributable to the diversity of participants, diagnostic criteria and approaches to data analysis. Inflammation status was rarely adequately assessed, and we conclude the burden of ID may well be under-recognised, particularly in children and young people living with overnutrition.

## Data Availability

All data relevant to the study are included in the article or uploaded as supplementary information.

## References

[1] Bailey RL, West KP, Black RE. The epidemiology of global Micronutrient deficiencies. Ann Nutr Metab 2015;66 Suppl 2(suppl 2):22–33. 10.1159/000371618 26045325

[2] Black RE, Victora CG, Walker SP, et al. Maternal and child Undernutrition and overweight in low-income and middle-income countries. Lancet 2013;382:427–51. 10.1016/S0140-6736(13)60937-X 23746772

[3] Tam E, Keats EC, Rind F, et al. Micronutrient supplementation and Fortification interventions on health and development outcomes among children under-five in Low- and middle-income countries: A systematic review and meta-analysis. Nutrients 2020;12:289. 10.3390/nu12020289 31973225 PMC7071447

[4] da Silva Lopes K, Yamaji N, Rahman MO, et al. Nutrition-specific interventions for preventing and controlling anaemia throughout the life cycle: an overview of systematic reviews. Cochrane Database Syst Rev 2021;9:CD013092. 10.1002/14651858.CD013092.pub2 34564844 PMC8464655

[5] Stevens GA, Beal T, Mbuya MNN, et al. Micronutrient deficiencies among preschool-aged children and women of reproductive age worldwide: a pooled analysis of individual-level data from population-representative surveys. Lancet Glob Health 2022;10:e1590–9. 10.1016/S2214-109X(22)00367-9 36240826 PMC10918648

[6] Yue T, Zhang Q, Li G, et al. Global burden of nutritional deficiencies among children under 5 years of age from 2010 to 2019. Nutrients 2022;14:2685. 10.3390/nu14132685 35807863 PMC9268233

[7] Camaschella C. Iron deficiency. Blood 2019;133:30–9. 10.1182/blood-2018-05-815944 30401704

[8] Gupta S, Brazier AKM, Lowe NM. Zinc deficiency in Low- and middle-income countries: prevalence and approaches for mitigation. J Hum Nutr Diet 2020;33:624–43. 10.1111/jhn.12791 32627912

[9] Belay A, Gashu D, Joy EJM, et al. Zinc deficiency is highly prevalent and spatially dependent over short distances in Ethiopia. Sci Rep 2021;11:6510. 10.1038/s41598-021-85977-x 33753836 PMC7985319

[10] Laillou A, Pham TV, Tran NT, et al. Micronutrient deficits are still public health issues among women and young children in Vietnam. PLoS One 2012;7:e34906. 10.1371/journal.pone.0034906 22529954 PMC3328495

[11] Sahile Z, Yilma D, Tezera R, et al. Prevalence of vitamin A deficiency among preschool children in Ethiopia: A systematic review and meta-analysis. Biomed Res Int 2020;2020:8032894. 10.1155/2020/8032894 32258145 PMC7073500

[12] Van Nhien N, Khan NC, Ninh NX, et al. Micronutrient deficiencies and anemia among preschool children in rural Vietnam. Asia Pac J Clin Nutr 2008;17:48–55. 18364326

[13] World Health Organization. Malnutrition. 2023. Available: https://www.who.int/health-topics/malnutrition#tab=tab_1 [Accessed 10 Mar 2023].

[14] Alderman H, Behrman JR, Glewwe P, et al. Child and adolescent health and development. In: Bundy DAP, Silva ND, Horton S, eds. Evidence of Impact of Interventions on Growth and Development during Early and Middle Childhood. Washington (DC): The International Bank for Reconstruction and Development / The World Bank©2017, 2017. 10.1596/978-1-4648-0423-6 30212122

[15] Astrup A, Bügel S. Overfed but Undernourished: recognizing nutritional inadequacies/deficiencies in patients with overweight or obesity. Int J Obes (Lond) 2019;43:219–32. 10.1038/s41366-018-0143-9 29980762

[16] Zhao L, Zhang X, Shen Y, et al. Obesity and iron deficiency: a quantitative meta-analysis. Obes Rev 2015;16:1081–93. 10.1111/obr.12323 26395622

[17] Gu K, Xiang W, Zhang Y, et al. The association between serum zinc level and overweight/obesity: a meta-analysis. Eur J Nutr 2019;58:2971–82. 10.1007/s00394-018-1876-x 30542939

[18] Saeed A, Dullaart RPF, Schreuder TCMA, et al. Disturbed vitamin A metabolism in non-alcoholic fatty liver disease (NAFLD). Nutrients 2017;10:29. 10.3390/nu10010029 29286303 PMC5793257

[19] Lowe NM. The global challenge of hidden hunger: perspectives from the field. Proc Nutr Soc 2021;80:283–9. 10.1017/S0029665121000902 33896431

[20] Poti JM, Braga B, Qin B. Ultra-processed food intake and obesity: what really matters for health-processing or nutrient content Curr Obes Rep 2017;6:420–31. 10.1007/s13679-017-0285-4 29071481 PMC5787353

[21] Moore JB. COVID-19, childhood obesity, and NAFLD: Colliding Pandemics. The Lancet Gastroenterology & Hepatology 2022;7:499–501. 10.1016/S2468-1253(22)00100-5 35550045 PMC9084622

[22] Popkin BM, Corvalan C, Grummer-Strawn LM. Dynamics of the double burden of malnutrition and the changing nutrition reality. The Lancet 2020;395:65–74. 10.1016/S0140-6736(19)32497-3 PMC7179702 31852602

[23] Di Cesare M, Sorić M, Bovet P, et al. The Epidemiological burden of obesity in childhood: a worldwide epidemic requiring urgent action. BMC Med 2019;17. 10.1186/s12916-019-1449-8 PMC6876113 31760948

[24] Castillo-Valenzuela O, Duarte L, Arredondo M, et al. Childhood obesity and plasma Micronutrient deficit of Chilean children between 4 and 14 years old. Nutrients 2023;15:1707. 10.3390/nu15071707 37049547 PMC10096594

[25] Tan PY, Mohd Johari SN, Teng K-T, et al. High prevalence of malnutrition and vitamin A deficiency among schoolchildren of rural areas in Malaysia using a multi-school assessment approach. Br J Nutr 2023;129:454–67. 10.1017/S0007114522001398 35506400

[26] Goyena E, Maniego MaL, Ducay AJ, et al. Dietary zinc intake and the underlying factors of serum zinc deficiency among preschool children in the Philippines. Philipp J Sci 2021;150:799–812. 10.56899/150.03.16

[27] Ssentongo P, Ba DM, Ssentongo AE, et al. Association of vitamin A deficiency with early childhood Stunting in Uganda: A population-based cross-sectional study. PLoS One 2020;15:e0233615. 10.1371/journal.pone.0233615 32470055 PMC7259702

[28] Cobayashi F, Augusto RA, Lourenço BH, et al. Factors associated with Stunting and overweight in Amazonian children: a population-based, cross-sectional study. Public Health Nutr 2014;17:551–60. 10.1017/S1368980013000190 23452910 PMC10282398

[29] Prentice AM. The triple burden of malnutrition in the era of globalization. Nestle Nutr Inst Workshop Ser 2023;97:51–61. 10.1159/000529005 37023735

[30] Malden S, Gillespie J, Hughes A, et al. Obesity in young children and its relationship with diagnosis of asthma, vitamin D deficiency, iron deficiency, specific allergies and flat-Footedness: A systematic review and meta-analysis. Obes Rev 2021;22:e13129. 10.1111/obr.13129 32808447 PMC7611974

[31] Fiamenghi VI, Mello ED de. Vitamin D deficiency in children and adolescents with obesity: a meta-analysis. Jornal de Pediatria 2021;97:273–9. 10.1016/j.jped.2020.08.006 33022267 PMC9432231

[32] Moher D, Liberati A, Tetzlaff J, et al. Reprint--preferred reporting items for systematic reviews and meta-analyses: the PRISMA statement. Phys Ther 2009;89:873–80. 19723669

[33] Sawyer SM, Azzopardi PS, Wickremarathne D, et al. The age of adolescence. Lancet Child Adolesc Health 2018;2:223–8. 10.1016/S2352-4642(18)30022-1 30169257

[34] Ouzzani M, Hammady H, Fedorowicz Z, et al. Rayyan—a web and mobile App for systematic reviews. Syst Rev 2016;5:210. 10.1186/s13643-016-0384-4 27919275 PMC5139140

[35] Academy of Nutrition and Dietetics. Evidence Analysis Manual: Academy of Nutrition and Dietetics. 2016. 10.1016/j.jand.2013.12.006 24439820

[36] Jackson D, Turner R. Power analysis for random-effects meta-analysis. Res Synth Methods 2017;8:290–302. 10.1002/jrsm.1240 28378395 PMC5590730

[37] Wickham H. Ggplot2. In: ggplot2: Elegant Graphics for Data Analysis. Cham: Springer-Verlag New York, 2016. 10.1007/978-3-319-24277-4

[38] Brunson JC. Ggalluvial: layered grammar for alluvial plots. J Open Source Softw 2020;5:49. 10.21105/joss.02017 PMC10010671 36919162

[39] South A. Rworldmap: a new R package for mapping global data. The R Journal 2011;3:35. 10.32614/RJ-2011-006

[40] R: A language and environment for statistical computing program. Vienna, Austria R Foundation for Statistical Computing; 2023.

[41] Park JS, Chang JY, Hong J, et al. Nutritional zinc status in Weaning infants: association with iron deficiency, age, and growth profile. Biol Trace Elem Res 2012;150:91–102. 10.1007/s12011-012-9509-3 23054863

[42] Lu J, Zhang H, Cao W, et al. Study on the zinc nutritional status and risk factors of Chinese 6-18-year-old children. Nutrients 2023;15:1685. 10.3390/nu15071685 37049525 PMC10096995

[43] Kernan KF, Carcillo JA. Hyperferritinemia and inflammation. Int Immunol 2017;29:401–9. 10.1093/intimm/dxx031 28541437 PMC5890889

[44] World Health Organization. WHO guideline on use of ferritin concentrations to assess iron status in individuals and populations. Geneva, 2020. 33909381

[45] Nead KG, Halterman JS, Kaczorowski JM, et al. Overweight children and adolescents: a risk group for iron deficiency. Pediatrics 2004;114:104–8. 10.1542/peds.114.1.104 15231915

[46] Ortíz Pérez M, Vázquez López MA, Ibáñez Alcalde M, et al. Relationship between obesity and iron deficiency in healthy adolescents. Child Obes 2020;16:440–7. 10.1089/chi.2019.0276 32877290

[47] Pompano LM, Correa-Burrows P, Burrows R, et al. Adjusting Ferritin concentrations for Nonclinical inflammation in adolescents with overweight or obesity. J Pediatr 2022;244:125–32. 10.1016/j.jpeds.2022.01.012 35074310

[48] Suteerojntrakool O, Khongcharoensombat T, Chomtho S, et al. Anthropometric markers and iron status of 6-12-year-old Thai children: associations and predictors. J Nutr Metab 2021;2021:9629718. 10.1155/2021/9629718 33953979 PMC8057914

[49] Ferrari M, Cuenca-García M, Valtueña J, et al. Inflammation profile in overweight/obese adolescents in Europe: an analysis in relation to iron status. Eur J Clin Nutr 2015;69:247–55. 10.1038/ejcn.2014.154 25205319

[50] Kurniawan YAI, Muslimatun S, Achadi EL, et al. Anaemia and iron deficiency anaemia among young adolescent girls from the peri urban Coastal area of Indonesia. Asia Pac J Clin Nutr 2006;15:350–6. 16837427

[51] Sethy PGS, Bulliyya G, Rautray TR, et al. Nutritional status of preschool children in association with some trace elements in rural gram Panchayatas of Bhubaneswar, Odisha, India. Adv Sci Lett 2014;20:868–73. 10.1166/asl.2014.5414

[52] Ghosh A, Chowdhury SD, Ghosh T. Undernutrition in Nepalese children: a biochemical and haematological study. Acta Paediatr 2012;101:671–6. 10.1111/j.1651-2227.2012.02613.x 22283707

[53] Brotanek JM, Gosz J, Weitzman M, et al. Iron deficiency in early childhood in the United States: risk factors and racial/ethnic disparities. Pediatrics 2007;120:568–75. 10.1542/peds.2007-0572 17766530

[54] Tussing-Humphreys LM, Liang H, Nemeth E, et al. Excess Adiposity, inflammation, and iron-deficiency in female adolescents. J Am Diet Assoc 2009;109:297–302. 10.1016/j.jada.2008.10.044 19167957

[55] Cepeda-Lopez AC, Osendarp SJ, Melse-Boonstra A, et al. Sharply higher rates of iron deficiency in obese Mexican women and children are predicted by obesity-related inflammation rather than by differences in dietary iron intake. Am J Clin Nutr 2011;93:975–83. 10.3945/ajcn.110.005439 21411619

[56] Cabañas Pujadas G, Ortiz-Marrón H, Ortiz-Pinto MA, et al. Changes in obesity and iron deficiency between 4 and 9 years of age. longitudinal study of childhood obesity (ELOIN). Int J Obes (Lond) 2022;46:1992–9. 10.1038/s41366-022-01196-y 35931811

[57] Manios Y, Moschonis G, Chrousos GP, et al. The double burden of obesity and iron deficiency on children and adolescents in Greece: the healthy growth study. J Hum Nutr Diet 2013;26:470–8. 10.1111/jhn.12025 23279448

[58] Moschonis G, Chrousos GP, Lionis C, et al. Association of total body and visceral fat mass with iron deficiency in Preadolescents: the healthy growth study. Br J Nutr 2012;108:710–9. 10.1017/S0007114511005952 22088365

[59] Higgins JT, Chandler J, Cumpston M, et al. Cochrane Handbook for systematic reviews of interventions version 6.4. Cochrane 2023.

[60] Eftekhari M, Mozaffari-Khosravi H, Shidfar F. The relationship between BMI and iron status in iron-deficient adolescent Iranian girls. Public Health Nutr 2009;12:2377–81. 10.1017/S1368980009005187 19278566

[61] Yalçın SS, Fırat MÇ, Tosun E, et al. A possible Etiological factor in obesity: element status in blood and tooth of overweight versus normal-weight children. Int J Environ Health Res 2018;2018:1–13. 10.1080/09603123.2018.1531115 30318909

[62] Freake HC, Sankavaram K. Zinc: physiology, dietary sources, and requirements. In: Caballero B, ed. Encyclopedia of Human Nutrition. Fourth Edition. Oxford: Academic Press, 2013: 584–92.

[63] Naupal-Forcadilla RT, Barba CV, Talavera MTM, et al. Determinants of zinc status of 2-3-year-old children in Laguna, Philippines. Malays J Nutr 2017;23.

[64] International Zinc Nutrition Consultative Group. Assessing population zinc status with serum zinc concentration. 2012. Available: https://static1.squarespace.com/static/56424f6ce4b0552eb7fdc4e8/t/5774378f414fb5410541b748/1467234199261/IZiNCG_TechBrief2_2012-3.pdf

[65] Lowe NM, Fekete K, Decsi T. Methods of assessment of zinc status in humans: a systematic review. Am J Clin Nutr 2009;89:2040S–2051S. 10.3945/ajcn.2009.27230G 19420098

[66] Thillan K, Lanerolle P, Thoradeniya T, et al. Micronutrient status and associated factors of Adiposity in primary school children with normal and high body fat in Colombo municipal area, Sri Lanka. BMC Pediatr 2021;21:14. 10.1186/s12887-020-02473-3 33407272 PMC7786904

[67] Zhu Q, Dai Y, Zhang J, et al. Association between serum zinc concentrations and metabolic risk factors among Chinese children and adolescents. Br J Nutr 2021;126:1529–36. 10.1017/S0007114521000258 33472712

[68] Fan Y, Zhang C, Bu J. Relationship between selected serum metallic elements and obesity in children and adolescent in the U.S. Nutrients 2017;9:104. 10.3390/nu9020104 28165362 PMC5331535

[69] Perrone L, Gialanella G, Moro R, et al. Zinc, copper, and iron in obese children and adolescents. Nutrition Research 1998;18:183–9. 10.1016/S0271-5317(98)00011-6

[70] Dehghani SM, Katibeh P, Haghighat M, et al. Prevalence of zinc deficiency in 3-18 years old children in Shiraz-Iran. Iran Red Crescent Med J 2011;13:4–8. 22946012 PMC3407579

[71] Habib A, Molayemat M, Habib A, et al. Vitamin D and zinc are interlinked but affected by different growth factors in Iranian children and adolescents: vitamin D and zinc in Iranian children and adolescents. Iran J Pediatr 2022;32. 10.5812/ijp-127158

[72] Sharif Y, Sadeghi O, Dorosty A, et al. Serum levels of vitamin D, Retinol and zinc in relation to overweight among toddlers: findings from a national study in Iran. Arch Iran Med 2019;22:174–81. 31126175

[73] Li W, Herrán OF, Villamor E. Trends in iron, zinc, and vitamin A status biomarkers among Colombian children: results from 2 nationally representative surveys. Food Nutr Bull 2017;38:146–57. 10.1177/0379572117700976 28359210

[74] Ho M, Baur LA, Cowell CT, et al. Zinc status, dietary zinc intake and metabolic risk in Australian children and adolescents; Nepean longitudinal study. Eur J Nutr 2017;56:2407–14. 10.1007/s00394-016-1280-3 27475431

[75] Zou Y, Zhang R, Huang L, et al. Serum levels of vitamin D, Retinol, zinc, and CRP in relation to obesity among children and adolescents. Eur J Med Res 2022;27:51. 10.1186/s40001-022-00670-7 35379317 PMC8977183

[76] Lin C-N, Wilson A, Church BB, et al. Pediatric reference intervals for serum copper and zinc. Clin Chim Acta 2012;413:612–5. 10.1016/j.cca.2011.12.005 22192859

[77] EFSA Panel on Dietetic Products, Nutrition and Allergies. Scientific opinion on dietary reference values for zinc. EFSA Journal 2014;12:10. 10.2903/j.efsa.2014.3844 PMC13136992 42087959

[78] Centers for Disease Control and Prevention, World Health Organization, Nutrition International, et al. Micronutrient survey manual. Geneva: World Health Organization, 2020.

[79] Gunanti IR, Marks GC, Al-Mamun A, et al. Low serum concentrations of carotenoids and vitamin E are associated with high Adiposity in Mexican-American children. J Nutr 2014;144:489–95. 10.3945/jn.113.183137 24500938

[80] Hu W, Tong S, Oldenburg B, et al. Serum vitamin A concentrations and growth in children and adolescents in Gansu province, China. Asia Pac J Clin Nutr 2001;10:63–6. 10.1046/j.1440-6047.2001.00208.x 11708611

[81] Ortega-Senovilla H, de Oya M, Garcés C. Relationship of NEFA concentrations to Rbp4 and to Rbp4/Retinol in Prepubertal children with and without obesity. J Clin Lipidol 2019;13:301–7. 10.1016/j.jacl.2019.01.006 30773418

[82] Tian T, Wang Y, Xie W, et al. Associations between serum vitamin A and metabolic risk factors among Eastern Chinese children and adolescents. Nutrients 2022;14:610. 10.3390/nu14030610 35276969 PMC8839095

[83] Yang C, Chen J, Liu Z, et al. Association of vitamin A status with Overnutrition in children and adolescents. Int J Environ Res Public Health 2015;12:15531–9. 10.3390/ijerph121214998 26690192 PMC4690934

[84] Disalvo L, Varea A, Matamoros N. Vitamin A deficiency and associated factors in Preschoolers from the outskirts of La Plata, Buenos Aires. Arch Argent Pediat 2019;117:19–25. 10.5546/aap.2019.eng.19 30652442

[85] Maslova E, Mora-Plazas M, Forero Y, et al. Are vitamin A and iron deficiencies re-emerging in urban Latin America? A survey of schoolchildren in Bogota, Colombia. Food Nutr Bull 2009;30:103–11. 10.1177/156482650903000201 19689088

[86] Abd-El Wahed MA, Mohamed MH, Ibrahim SS, et al. Iron profile and dietary pattern of primary school obese Egyptian children. J Egypt Public Health Assoc 2014;89:53–9. 10.1097/01.EPX.0000451827.84315.5c 25162735

[87] de Souza Valente da Silva L, Valeria da Veiga G, Ramalho RA. Association of serum concentrations of Retinol and carotenoids with overweight in children and adolescents. Nutrition 2007;23:392–7. 10.1016/j.nut.2007.02.009 17433621

[88] Paes-Silva RP, Gadelha PCFP, Lemos M da CC de, et al. Adiposity, inflammation and fat-soluble vitamins in adolescents. Jornal de Pediatria 2019;95:575–83. 10.1016/j.jped.2018.05.008 29963989

[89] Dallazen C, Tietzmann DC, da Silva SA, et al. Vitamin A deficiency and associated risk factors in children aged 12-59 months living in poorest municipalities in the South region of Brazil. Public Health Nutr 2023;26:132–42. 10.1017/S1368980022000325 35125127 PMC11077461

[90] Wei X, Peng R, Cao J, et al. Serum vitamin A status is associated with obesity and the metabolic syndrome among school-age children in Chongqing, China. Asia Pac J Clin Nutr 2016;25:563–70. 10.6133/apjcn.092015.03 27440692

[91] Hutchinson C. A review of iron studies in overweight and obese children and adolescents: a double burden in the young Eur J Nutr 2016;55:2179–97. 10.1007/s00394-016-1155-7 26883916

[92] González-Domínguez Á, Visiedo-García FM, Domínguez-Riscart J, et al. Iron metabolism in obesity and metabolic syndrome. Int J Mol Sci 2020;21:15. 10.3390/ijms21155529 PMC7432525 32752277

[93] Haschka D, Hoffmann A, Weiss G. Iron in immune cell function and host defense. Semin Cell Dev Biol 2021;115:27–36. 10.1016/j.semcdb.2020.12.005 33386235

[94] Weiss G, Ganz T, Goodnough LT. Anemia of inflammation. Blood 2019;133:40–50. 10.1182/blood-2018-06-856500 30401705 PMC6536698

[95] Berton PF, Gambero A. Hepcidin and inflammation associated with iron deficiency in childhood obesity - A systematic review. J Pediatr (Rio J) 2024;100:124–31. 10.1016/j.jped.2023.06.002 37541648 PMC10943301

[96] Coimbra S, Catarino C, Nascimento H, et al. Physical exercise intervention at school improved Hepcidin, inflammation, and iron metabolism in overweight and obese children and adolescents. Pediatr Res 2017;82:781–8. 10.1038/pr.2017.139 28604755

[97] Amato A, Santoro N, Calabrò P, et al. Effect of body mass index reduction on serum Hepcidin levels and iron status in obese children. Int J Obes (Lond) 2010;34:1772–4. 10.1038/ijo.2010.204 20877286

[98] Zimmermann MB, Zeder C, Muthayya S, et al. Adiposity in women and children from transition countries predicts decreased iron absorption, iron deficiency and a reduced response to iron Fortification. Int J Obes (Lond) 2008;32:1098–104. 10.1038/ijo.2008.43 18427564

[99] Perng W, Mora-Plazas M, Marin C, et al. Iron status and linear growth: a prospective study in school-age children. Eur J Clin Nutr 2013;67:646–51. 10.1038/ejcn.2013.56 23462945

[100] Zhu Y, He B, Xiao Y, et al. Iron metabolism and its association with Dyslipidemia risk in children and adolescents: a cross-sectional study. Lipids Health Dis 2019;18:50. 10.1186/s12944-019-0985-8 30755213 PMC6371579

[101] Cheng HL, Bryant CE, Rooney KB, et al. Iron, Hepcidin and inflammatory status of young healthy overweight and obese women in Australia. PLoS One 2013;8:e68675. 10.1371/journal.pone.0068675 23861932 PMC3701675

[102] Onabanjo OO, Balogun OL. Anthropometric and iron status of adolescents from selected secondary schools in Ogun state. ICAN: Infant, Child, & Adolescent Nutrition 2014;6:109–18. 10.1177/1941406414520703

[103] Kassem E, Na’amnih W, Shapira M, et al. Comparison between school-age children with and without obesity in nutritional and inflammation biomarkers. J Clin Med 2022;11:23. 10.3390/jcm11236973 PMC9739253 36498548

[104] Shattnawi KK, Alomari MA, Al-Sheyab N, et al. The relationship between plasma Ferritin levels and body mass index among adolescents. Sci Rep 2018;8:15307. 10.1038/s41598-018-33534-4 30333502 PMC6193036

[105] Sypes EE, Parkin PC, Birken CS, et al. Higher body mass index is associated with iron deficiency in children 1 to 3 years of age. J Pediatr 2019;207:198–204. 10.1016/j.jpeds.2018.11.035 30630632

[106] Pasricha S-R, Tye-Din J, Muckenthaler MU, et al. Iron deficiency. The Lancet 2021;397:233–48. 10.1016/S0140-6736(20)32594-0 33285139

[107] El Khoury R, Sleilaty G, Gannagé-Yared M-H. Prevalence of iron deficiency in Lebanese schoolchildren. Eur J Clin Nutr 2020;74:1157–63. 10.1038/s41430-020-0590-y 32127687

[108] Tessema M, De Groote H, D. Brouwer I, et al. Soil zinc is associated with serum zinc but not with linear growth of children in Ethiopia. Nutrients 2019;11:221. 10.3390/nu11020221 30678175 PMC6413067

[109] Araújo LKAR de, Faria JCP, Sarni ROS. Iron deficiency anemia in infants in Sousa (PB), Brazil: an association with nutritional status. Rev Assoc Med Bras 2022;68:1698–704. 10.1590/1806-9282.20220761 36477102 PMC9779981

[110] Mei Z, Addo OY, Jefferds ME, et al. Physiologically based serum Ferritin thresholds for iron deficiency in children and non-pregnant women: a US national health and nutrition examination surveys (NHANES) serial cross-sectional study. The Lancet Haematology 2021;8:e572–82. 10.1016/S2352-3026(21)00168-X 34329578 PMC8948503

[111] EFSA Panel on Dietetic Products, Nutrition and Allergies. Scientific opinion on dietary reference values for vitamin A. EFSA Journal 2015;13:4028. 10.2903/j.efsa.2015.4028 PMC13151645 42109809

[112] Thurnham DI, McCabe LD, Haldar S, et al. Adjusting plasma Ferritin concentrations to remove the effects of Subclinical inflammation in the assessment of iron deficiency: a meta-analysis. Am J Clin Nutr 2010;92:546–55. 10.3945/ajcn.2010.29284 20610634

[113] Larson LM, Guo J, Williams AM, et al. Approaches to assess vitamin A status in settings of inflammation: biomarkers reflecting inflammation and nutritional determinants of anemia (BRINDA) project. Nutrients 2018;10:1100. 10.3390/nu10081100 30115830 PMC6115742

[114] McDonald CM, Suchdev PS, Krebs NF, et al. Adjusting plasma or serum zinc concentrations for inflammation: biomarkers reflecting inflammation and nutritional determinants of anemia (BRINDA) project. Am J Clin Nutr 2020;111:927–37. 10.1093/ajcn/nqz304 32266402 PMC7138668

[115] Namaste SM, Aaron GJ, Varadhan R, et al. Methodologic approach for the biomarkers reflecting inflammation and nutritional determinants of anemia (BRINDA) project. Am J Clin Nutr 2017;106:Suppl. 10.3945/ajcn.116.142273 PMC5490643 28615254

[116] Namaste SM, Rohner F, Huang J, et al. Adjusting Ferritin concentrations for inflammation: biomarkers reflecting inflammation and nutritional determinants of anemia (BRINDA) project. Am J Clin Nutr 2017;106(Suppl 1):359S–371S. 10.3945/ajcn.116.141762 28615259 PMC5490647

[117] UNICEF, WHO. World Bank Group Joint Child Malnutrition Estimates. Levels and Trends in Child Malnutrition. 2018.

[118] Borkhoff SA, Parkin PC, Birken CS, et al. Examining the double burden of Underweight, overweight/obesity and iron deficiency among young children in a Canadian primary care setting. Nutrients 2023;15:3635. 10.3390/nu15163635 37630825 PMC10458882

[119] Aslam S, Emmanuel P. Formulating a researchable question: A critical step for facilitating good clinical research. Indian J Sex Transm Dis AIDS 2010;31:47–50. 10.4103/0253-7184.69003 21808439 PMC3140151

