# Supplementary material for: Overnutrition is a risk factor for iron, but not for zinc or vitamin A deficiency in children and young people: a systematic review and meta-analysis
Source: BMJ Glob Health. 2024 Apr 9;9(4):e015135. doi: 10.1136/bmjgh-2024-015135 (PMC11015307; doi:10.1136/bmjgh-2024-015135)
Supplement: Supplementary data [file bmjgh-2024-015135supp001.pdf]

**Tan *et al.* Supplementary Material**

## SUPPLEMENTARY MATERIAL

Overnutrition is a risk factor for iron, but not zinc or vitamin A deficiency in children and young people: a systematic review and meta-analysis

### Authors

Tan X, Tan PY, Gong YY and Moore JB[1].

[1]Corresponding Author: J.B.Moore@leeds.ac.uk

### Table of Contents

Table S1 Search strategy for Medline. ....................2

Table S2 Search strategy for Embase. ....................4

Table S3 Search strategy for Scopus. ....................6

Table S4 Search strategy for Cochrane. ....................8

Table S5 Characteristics of studies (n=46) assessing the association between iron and under- and overnutrition. ....................10

Table S6 Characteristics of the studies (n=28) assessing the association between zinc and weight status. ....................17

Table S7 Characteristics of the studies (n=27) assessing the association between vitamin A and weight status. ....................20

Table S8 Risk of bias assessment for included studies. ....................24

Figure S1 Subgroup analysis of associations between iron deficiency and overnutrition stratified by gender. ....................27

Figure S2 Funnel plots for nutrition status and gender. ....................28

Figure S3 Leave-one-out sensitivity test for overall and subgroup effect sizes. ....................29

## Tan *et al.* Supplementary Material

**Table S1 Search strategy for Medline.**

| # ▲ | Searches | Results |
|---|---|---|
| 1 | Anaemia, Iron-Deficiency/bl, di, ep, et, me [Blood, Diagnosis, Epidemiology, Etiology, Metabolism] | 7101 |
| 2 | Iron/bl, df [Blood, Deficiency] | 17580 |
| 3 | ((iron or ferritin or hemoglobin) and (serum or blood or plasma) and (inadequa* or insufficien* or deficien*)).ti,ab,kw. | 16221 |
| 4 | 1 or 2 or 3 | 33690 |
| 5 | Zinc/bl, df [Blood, Deficiency] | 11105 |
| 6 | (zinc and (serum or blood or plasma) and (inadequa* or insufficien* or deficien*)).ti,ab,kw. | 4505 |
| 7 | 5 or 6 | 13387 |
| 8 | Vitamin A Deficiency/bl, di, ep, et, pc [Blood, Diagnosis, Epidemiology, Etiology, Prevention & Control] | 2240 |
| 9 | Vitamin A/bl [Blood] | 5182 |
| 10 | ((retinol or "vitamin A") and (serum or blood or plasma) and (inadequa* or insufficien* or deficien*)).ti,ab,kw. | 2923 |
| 11 | 8 or 9 or 10 | 8055 |
| 12 | 4 or 7 or 11 | 51425 |
| 13 | Obesity/bl, di, ep, et, me, pc [Blood, Diagnosis, Epidemiology, Etiology, Metabolism, Prevention & Control] | 98565 |
| 14 | Obesity, Abdominal/bl, di, ep, me, pc [Blood, Diagnosis, Epidemiology, Metabolism, Prevention & Control] | 2530 |
| 15 | Pediatric Obesity/bl, di, ep, et, me, pc [Blood, Diagnosis, Epidemiology, Etiology, Metabolism, Prevention & Control] | 6908 |
| 16 | Overweight/bl, di, ep, et, me, pc [Blood, Diagnosis, Epidemiology, Etiology, Metabolism, Prevention & Control] | 13827 |
| 17 | Malnutrition/bl, di, ep, et, me, pc [Blood, Diagnosis, Epidemiology, Etiology, Metabolism, Prevention & Control] | 9368 |
| 18 | Protein-Energy Malnutrition/bl, di, ep, et, me, pc [Blood, Diagnosis, Epidemiology, Etiology, Metabolism, Prevention & Control] | 4276 |
| 19 | Thinness/bl, di, ep, et, pc [Blood, Diagnosis, Epidemiology, Etiology, Prevention & Control] | 2585 |
| 20 | Body Mass Index/ | 130582 |
| 21 | Anthropometry/ | 39336 |
| 22 | (malnutrition or malnourish* or overnutrition or undernutrition or obese or obesity or overweight or adiposity or stunting or stunted or underweight or (wasting adj2 disease) or wasted or "body mass index" or BMI or anthropometr*).ti,ab,kw. | 586872 |
| 23 | 13 or 14 or 15 or 16 or 17 or 18 or 19 or 20 or 21 or 22 | 644487 |
| 24 | 12 and 23 | 4893 |
| 25 | limit 24 to english | 4565 |
| 26 | exp adolescent/ or exp child/ or exp infant/ or (infant disease* or childhood disease*).ti,ab,kf. or (adolescen* or babies or baby or boy? or boyfriend or boyhood or girlfriend or girlhood or child* or girl? or infan* or juvenil* or kid? or minors or minors* or neonat* or neo-nat* or newborn* or new-born* or paediatric* or peadiatric* or pediatric* or perinat* or preschool* or puber* or pubescen* or school* or teen* or toddler? or underage? or under-age? or youth*).ti,ab,kf. or (pediatric* or | 5094920 |



Tan X, *et al. BMJ Glob Health* 2024; 9:e015135. doi: 10.1136/bmjgh-2024-015135

**Tan *et al.* Supplementary Material**

| | | |
|---|---|---|
| | paediatric* or infan* or child* or adolescen* or young).jn,jw. or (pediatric* or paediatric* or infan* or child* or adolescen* or young).in. | |
| 27 | 25 and 26 | 2549 |
| 28 | limit 27 to (address or case reports or comment or editorial or letter or meta analysis or news or "review" or "systematic review") | 302 |
| 29 | 27 not 28 | 2247 |



Tan X, *et al. BMJ Glob Health* 2024; 9:e015135. doi: 10.1136/bmjgh-2024-015135

**Tan *et al.* Supplementary Material**

4 **Table S2 Search strategy for Embase.**

| # ▲ | Searches | Results |
|---|---|---|
| 1 | iron/ec [Endogenous Compound] | 20090 |
| 2 | iron deficiency/co, di, dm, ep, et, pc [Complication, Diagnosis, Disease Management, Epidemiology, Etiology, Prevention] | 2292 |
| 3 | iron deficiency anaemia/co, di, dm, ep, et, pc [Complication, Diagnosis, Disease Management, Epidemiology, Etiology, Prevention] | 6259 |
| 4 | ((iron or ferritin or hemoglobin) and (serum or blood or plasma) and (inadequa* or insufficien* or deficien*)).ti,ab,kw. | 24033 |
| 5 | or/1-4 | 46391 |
| 6 | zinc/ec [Endogenous Compound] | 8578 |
| 7 | zinc deficiency/co, di, ep, et, pc [Complication, Diagnosis, Epidemiology, Etiology, Prevention] | 825 |
| 8 | (zinc and (serum or blood or plasma) and (inadequa* or insufficien* or deficien*)).ti,ab,kw. | 5061 |
| 9 | or/6-8 | 12886 |
| 10 | retinol/ec [Endogenous Compound] | 4437 |
| 11 | retinol deficiency/co, di, ep, et, pc [Complication, Diagnosis, Epidemiology, Etiology, Prevention] | 1424 |
| 12 | ((retinol or "vitamin A") and (serum or blood or plasma) and (inadequa* or insufficien* or deficien*)).ti,ab,kw. | 2905 |
| 13 | or/10-12 | 7461 |
| 14 | 5 or 9 or 13 | 60740 |
| 15 | overnutrition/di, dm, ep, et, pc [Diagnosis, Disease Management, Epidemiology, Etiology, Prevention] | 234 |
| 16 | obesity/di, dm, ep, et, pc [Diagnosis, Disease Management, Epidemiology, Etiology, Prevention] | 47924 |
| 17 | abdominal obesity/di, dm, ep, et, pc [Diagnosis, Disease Management, Epidemiology, Etiology, Prevention] | 1226 |
| 18 | childhood obesity/di, dm, ep, et, pc [Diagnosis, Disease Management, Epidemiology, Etiology, Prevention] | 3482 |
| 19 | malnutrition/di, dm, ep, et, pc [Diagnosis, Disease Management, Epidemiology, Etiology, Prevention] | 7850 |
| 20 | protein calorie malnutrition/di, dm, ep, et, pc [Diagnosis, Disease Management, Epidemiology, Etiology, Prevention] | 1032 |
| 21 | underweight/di, dm, ep, et, pc [Diagnosis, Disease Management, Epidemiology, Etiology, Prevention] | 789 |
| 22 | stunting/di, dm, ep, et, pc [Diagnosis, Disease Management, Epidemiology, Etiology, Prevention] | 355 |
| 23 | chronic wasting disease/di, dm, ep, et, pc [Diagnosis, Disease Management, Epidemiology, Etiology, Prevention] | 234 |
| 24 | body mass index/ | 404144 |
| 25 | anthropometry/ | 47377 |
| 26 | (malnutrition or malnourish* or overnutrition or undernutrition or obese or obesity or overweight or adiposity or stunting or stunted or underweight or (wasting adj2 disease) or wasted or "body mass index" or BMI or anthropometr*).ti,ab,kw. | 859208 |
| 27 | or/15-26 | 961836 |
| 28 | 14 and 27 | 7182 |
| 29 | limit 28 to english | 6810 |

**Tan *et al.* Supplementary Material**

| | | |
|---|---|---|
| 30 | exp adolescence/ or exp adolescent/ or exp child/ or exp childhood disease/ or exp infant disease/ or (adolescen* or babies or baby or boy? or boyfriend or boyhood or girlfriend or girlhood or child* or girl? or infan* or juvenil* or juvenile* or kid? or minors or minors* or neonat* or neo-nat* or neo-nat* or newborn* or new-born* or paediatric* or peadiatric* or pediatric* or perinat* or preschool* or puber* or pubescen* or school or school child* or school* or schoolchild* or schoolchild* or pediatric* or paediatric* or infan* or child* or adolescen* or young).jn,jw. or (pediatric* or paediatric* or infan* or child* or adolescen* or young).in. or (teen* or toddler? or underage? or under-age? or youth*).ti,ab,kw. | 4255302 |
| 31 | 29 and 30 | 2812 |
| 32 | limit 31 to conference abstracts | 541 |
| 33 | limit 31 to (book or book series) | 10 |
| 34 | limit 31 to (books or chapter or conference abstract or letter or note or "review" or short survey) | 774 |
| 35 | limit 31 to (meta analysis or "systematic review") | 38 |
| 36 | 32 or 33 or 34 or 35 | 794 |
| 37 | 31 not 36 | 2018 |

**Tan *et al.* Supplementary Material**

**Table S3 Search strategy for Scopus.**

| # | Searching words | Results |
|---|---|---|
| #1 | TITLE-ABS-KEY ( ( iron OR ferritin OR hemoglobin OR zinc OR "vitamin A" OR retinol ) AND ( serum OR blood OR plasma ) AND ( inadequa* OR insufficien* OR deficien* ) ) | 60369 |
| #2 | TITLE-ABS-KEY ( malnutrition OR malnourish* OR overnutrition OR undernutrition OR obese OR obesity OR overweight OR adiposity OR stunting OR stunted OR underweight OR ( wasting W/2 disease ) OR wasted OR "body mass index" OR bmi OR anthropometr* ) | 887265 |
| #3 | ( TITLE-ABS-KEY ( ( iron OR ferritin OR hemoglobin OR zinc OR "vitamin W/0 A" OR retinol ) AND ( serum OR blood OR plasma ) AND ( inadequa* OR insufficien* OR deficien* ) ) ) AND ( TITLE-ABS ( malnutrition OR malnourish* OR overnutrition OR undernutrition OR obese OR obesity OR overweight OR adiposity OR stunting OR stunted OR underweight OR ( wasting W/2 disease ) OR wasted OR "body mass index" OR bmi OR anthropometr* ) ) | 7,726 |
| #4 | ( TITLE-ABS-KEY ( ( iron OR ferritin OR hemoglobin OR zinc OR "vitamin W/0 A" OR retinol ) AND ( serum OR blood OR plasma ) AND ( inadequa* OR insufficien* OR deficien* ) ) ) AND ( TITLE-ABS-KEY ( malnutrition OR malnourish* OR overnutrition OR undernutrition OR obese OR obesity OR overweight OR adiposity OR stunting OR stunted OR underweight OR ( wasting W/2 disease ) OR wasted OR "body mass index" OR bmi OR anthropometr* ) ) AND ( LIMIT-TO ( LANGUAGE , "English" ) ) | 7,173 |
| #5 | ( TITLE-ABS-KEY ( ( iron OR ferritin OR hemoglobin OR zinc OR "vitamin W/0 A" OR retinol ) AND ( serum OR blood OR plasma ) AND ( inadequa* OR insufficien* OR deficien* ) ) ) AND ( TITLE-ABS-KEY ( malnutrition OR malnourish* OR overnutrition OR undernutrition OR obese OR obesity OR overweight OR adiposity OR stunting OR stunted OR underweight OR ( wasting W/2 disease ) OR wasted OR "body mass index" OR bmi OR anthropometr* ) ) AND ( ( TITLE-ABS-KEY ( adolescen* OR babies OR baby OR boy? OR boyfriend OR boyhood OR girlfriend OR girlhood OR child* OR girl? OR infan* OR juvenil* OR kid? OR minors OR minors* OR neonat* OR neo-nat* OR newborn* OR new-born* OR paediatric* OR peadiatric* OR pediatric* OR perinat* OR preschool* OR puber* OR pubescen* OR school* OR teen* OR toddler? OR underage? OR under-age? OR youth* ) ) ) AND ( LIMIT-TO ( LANGUAGE , "English" ) ) | 3,036 |
| #6 | ( TITLE-ABS-KEY ( ( iron OR ferritin OR hemoglobin OR zinc OR "vitamin W/0 A" OR retinol ) AND ( serum OR blood OR plasma ) AND ( inadequa* OR insufficien* OR deficien* ) ) ) AND ( TIT | 2,683 |

**Tan *et al.* Supplementary Material**

| | | |
|---|---|---|
| | LE-ABS-<br>KEY ( malnutrition OR malnourish* OR overnutrition OR undernutrition OR obese OR obesity OR overweight OR a<br>diposity OR stunting OR stunted OR underweight OR ( wasting W/2 disease ) OR wasted OR "body mass<br>index" OR bmi OR anthropometr* ) ) AND ( ( TITLE-ABS-<br>KEY ( adolescen* OR babies OR baby OR boy? OR boyfriend OR boyhood OR girlfriend OR girlhood OR child*<br>OR girl? OR infan* OR juvenil* OR kid? OR minors OR minors* OR neonat* OR neo-<br>nat* OR newborn* OR new-<br>born* OR paediatric* OR peadiatric* OR pediatric* OR perinat* OR preschool* OR puber* OR pubescen* OR scho<br>ol* OR teen* OR toddler? OR underage? OR under-age? OR youth* ) ) ) AND ( LIMIT-<br>TO ( LANGUAGE , "English" ) ) AND ( LIMIT-TO ( DOCTYPE , "ar" ) ) | |

**Tan *et al.* Supplementary Material**

**Table S4 Search strategy for Cochrane.**

| | | |
|---|---|---|
| #1 | MeSH descriptor: [Iron, Dietary] explode all trees and with qualifier(s): [blood - BL] | 54 |
| #2 | MeSH descriptor: [Iron] explode all trees and with qualifier(s): [blood - BL, deficiency - DF] | 933 |
| #3 | (((iron or ferritin or hemoglobin) and (serum or blood or plasma)) and (inadequa* or insufficien* or deficien*)):ti,ab,kw | 5477 |
| #4 | MeSH descriptor: [Anaemia, Iron-Deficiency] explode all trees | 1330 |
| #5 | #1 or #2 or #3 or #4 | 6310 |
| #6 | MeSH descriptor: [Zinc] explode all trees and with qualifier(s): [blood - BL, deficiency - DF] | 648 |
| #7 | (((zinc) and (serum or blood or plasma)) and (inadequa* or insufficien* or deficien*)):ti,ab,kw | 870 |
| #8 | #6 or #7 | 1243 |
| #9 | MeSH descriptor: [Vitamin A] tree(s) exploded and with qualifier(s): [blood - BL] | 497 |
| #10 | (((retinol or "vitamin A") and (serum or blood or plasma)) and (inadequa* or insufficien* or deficien*)):ti,ab,kw | 706 |
| #11 | #9 or #10 | 977 |
| #12 | #5 or #8 or #11 | 7724 |
| #13 | MeSH descriptor: [Obesity] explode all trees | 14007 |
| #14 | MeSH descriptor: [Thinness] explode all trees and with qualifier(s): [blood - BL, diagnosis - DI, etiology - ET, metabolism - ME, epidemiology - EP, prevention & control - PC] | 111 |
| #15 | MeSH descriptor: [Malnutrition] explode all trees and with qualifier(s): [blood - BL, diagnosis - DI, etiology - ET, metabolism - ME, epidemiology - EP, prevention & control - PC] | 1969 |
| #16 | MeSH descriptor: [Overweight] explode all trees and with qualifier(s): [blood - BL, diagnosis - DI, etiology - ET, metabolism - ME, epidemiology - EP, prevention & control - PC] | 6395 |
| #17 | MeSH descriptor: [Protein-Energy Malnutrition] explode all trees | 250 |
| #18 | MeSH descriptor: [Body Mass Index] explode all trees | 10188 |
| #19 | MeSH descriptor: [Anthropometry] explode all trees | 23419 |
| #20 | (malnutrition or malnourish* or overnutrition or undernutrition or obese or obesity or overweight or adiposity or stunting or stunted or underweight or (wasting NEAR/2 disease) or wasted or "body mass index" or BMI or anthropometr*):ti,ab,kw | 99958 |
| #21 | #13 or #14 or #15 or #16 or #17 or #18 or #19 or #20 | 109600 |
| #22 | #12 and #21 | 1831 |
| #23 | MeSH descriptor: [Adolescent] explode all trees | 104274 |
| #24 | MeSH descriptor: [Child] explode all trees | 56347 |
| #25 | MeSH descriptor: [Infant] explode all trees | 32245 |
| #26 | (infant disease* or childhood disease*):ti,ab,kw or (adolescen* or babies or baby or boy? or boyfriend or boyhood or girlfriend or girlhood or child* or girl? or infan* or juvenil* or kid? or minors or minors* or neonat* or neo-nat* or newborn* or new-born* | 308863 |

**Tan *et al.* Supplementary Material**

| | | |
|---|---|---|
| | or paediatric* or peadiatric* or pediatric* or perinat* or preschool* or puber* or pubescen* or school* or teen* or toddler? or underage? or under-age? or youth*):ti,ab,kw | |
| #27 | #23 or #24 or #25 or #26 | 308863 |
| #28 | #22 and #27 in trials | 882 |

## Tan *et al.* Supplementary Material

13 **Table S5 Characteristics of studies (n=46) assessing the association between iron and under- and overnutrition.**

| First Author,<br>Year,<br>Country | Study Design,<br>Participant (n),<br>Age | Malnutrition and Biochemical Indicators[1] | Findings[2] | Association | Quality Rating |
|---|---|---|---|---|---|
| **Both (n=10)** | | | | | |
| Cobayashi,<br>2013,<br>Brazil[1] | Cross-sectional,<br>1139,<br>0-10 y | OW: BAZ>1; stunting<br>ID: SF<12μg/L (<5 y), <15μg/L (≥5 y), or sTfR>8.3mg/L | **Overnutrition**:<br>BAZ↑ ID↑ OW/ID: PR= 1.64 (1.07, 2.53) for <5 y<br>**Undernutrition:** NS | Inverse<br><br>NS | Positive |
| El Khoury,<br>2020,<br>Lebanon[2] | Cross-sectional,<br>903,<br>8-18 y | BMI category<br>ID: SF<15ng/mL; CRP>10mg/L excluded | **Both over- and undernutrition:** NS | NS | Neutral |
| Eftekhari,<br>2009,<br>Iran[3] | Cross-sectional,<br>431,<br>13-20 y | BMI category<br>ID: SF<12μg/L, TS<16% | **Both over- and undernutrition:**<br>BMI↑ SF↓ β= -0.21 for girls with ID | Inverse | Neutral |
| Li,<br>2017,<br>Colombia[4] | Cross-sectional,<br>14559,<br>1-17 y | HAZ, BAZ<br>PF | **Both over- and undernutrition:** NS | NS | Positive |
| Maslova,<br>2009,<br>Colombia[5] | Cross-sectional,<br>2811,<br>5-12 y | BAZ<br>ID: PF<15 or 30μg/L if CRP>10mg/L | **Both over- and undernutrition:**<br>PF↓ BAZ↓ ID/BAZ: β= -0.22 (-0.42, -0.03) | Direct | Positive |
| Onabanjo,<br>2014,<br>Nigeria[6] | Cross-sectional,<br>127,<br>10-19 y | Underweight: BAZ<5th centile<br>OW, OB: BAZ>85th & 95th centiles<br>SF | **Overnutrition**:<br>BMI↑ SF↑ OW, OB: r= 0.505, 0.556 (male); OW, OB: r= 0.782, 0.838 (female)<br>**Undernutrition**: NS | Direct<br><br>NS | Neutral |

**Tan *et al.* Supplementary Material**

| | | | | | |
|---|---|---|---|---|---|
| Perng,<br>2013,<br>Colombia[7] | Cohort study,<br>2714,<br>5-12y | BAZ, HAZ<br>PF | **Overnutrition:**<br>BAZ↑ PF↑<br>**Undernutrition:** NS | Direct<br><br>NS | Positive |
| Tan,<br>2023,<br>Malaysia[8] | Cross-sectional,<br>776,<br>7-11y | BAZ, WAZ, HAZ, stunting<br>ID: SF<15μg/L; Anaemia: Hb<115g/L;<br>IDA: ID + Anaemia | **Overnutrition**:<br>BAZ↑ SF↑; WAZ↑ SF↑<br>**Undernutrition:**<br>SF↓ HAZ↓ ID/stunting COR= 2.4 (1.6, 3.7)<br>SF, Hb↓ HAZ↓ IDA/stunting AOR= 2.3 (1.2, 4.3) | Direct | Positive |
| Zhu,<br>2019,<br>China[9] | Cross-sectional,<br>1866,<br>7-18 y | BMI category (Working Group on Obesity in China)<br>SF, SI<br>ID: SI<45μg/dL | **Overnutrition**:<br>BMI↑ Prevalence of ID ↑ SI↓<br>BMI↑ SF↑<br>**Undernutrition:** NS | Inverse<br><br><br>NS | Neutral |
| Zimmermann,<br>2008,<br>Morocco, India[10] | Cross-sectional,<br>1688,<br>9.3±2.5y | BAZ<br>SF, TfR<br>BIS (SF/TfR) | **Both over- and undernutrition:**<br>BAZ↑ BIS↓ y= -0.6256x+3.194; β= -0.658 (-0.466, -0.850)<br>BAZ↑ SF↑ β= 1.873 (1.587, 2.519)<br>BAZ↑ sTfR↑ β= 1.873 (1.587, 2.159) | Inverse | Neutral |
| **Overnutrition (n=21)** | | | | | |
| Abd-El-Wahed,<br>2014,<br>Egypt[11] | Case-control,<br>120 (60+60),<br>6-12 y | NW, OB: BMI<85th & >95th centiles<br>ID: ≥2 abnormal parameters: MCV<76fL, TS<15%, SF<10μg/mL | BMI↑ ID↑ ID/OB: COR= 7.09 (3.16, 15.92) | Inverse | Neutral |
| Brotanek,<br>2007,<br>US[12] | Cross-sectional,<br>1641,<br>1-3 y | OW, OB: WHZ>85th & 95th centiles<br>ID: ≥2 abnormal parameters: TS<10%, SF<10μg/L, red blood cells EP>1.42μmol/L (1-2 y); <12%, 10μmol/L, and>1.24μmol/L (3y) | WHZ↑ ID↑ ID/OW: AOR= 3.34 (1.10, 10.12) | Inverse | Positive |
| Cabañas Pujadas,<br>2022,<br>Spain[13] | Cohort study,<br>1347,<br>9 y | OB: BAZ>2<br>SF, Serum transferrin, CRP<br>ID: TS<16% | BAZ↑ TS↓ β= −2.4 (−4.6, −0.1)<br>BAZ↑ SF↑ β= 13.9 (7.2, 20.5)<br>BAZ↑ serum transferrin↑ β= 12.0 (3.9, 20.1) | Inverse | Positive |



Tan X, *et al. BMJ Glob Health* 2024; 9:e015135. doi: 10.1136/bmjgh-2024-015135

**Tan *et al.* Supplementary Material**

| | | | | | |
|---|---|---|---|---|---|
| Cepeda-Lopez, 2011, Mexico[14] | Cross-sectional, 1174, 5-12 y | OB, OW: BMIz>1 & 2 SD<br>TIBC, CRP<br>ID: low SI or TIBC >36 μg/L; TS<20%. | BMIz↑ ID↑ ID/OB: AOR= 3.96 (1.34, 11.67)<br>BMIz↑ TIBC↑ r= 0.04 | Inverse | Positive |
| Cheng, 2013, Australia[15] | Cross-sectional, 114, 18-25 y | BMI≥27.5<br>SI, TS, SF | **in OW/OB women**<br>BMI↑ SI↓ β= -0.379 (0.139)<br>BMI↑ TS↓ β= -0.588 (0.222)<br>BMI↑ SF↑ | Inverse | Positive |
| de Araújo, 2022, Brazil[16] | Cross-sectional, 104, 7-9 m | WHZ<br>ID: Hb>11g/dL, ferritin<12μg/L if CRP≤5mg/L, or 30μg/L if CRP>5mg/L | WHZ↑ ID↑ WHZ/ID: AOR= 2.86 (1.38, 5.6);<br>BAZ, WHZ, WAZ↑, ferritin↓ | Inverse | Neutral |
| Ferrari, 2015, Europe[317] | Cross-sectional, 876, 13.9-16 y | Thinness: BMI<18; OW, OB: BMI>25 & 30<br>SF, sTfR, CRP | BMI↑ SF↑ male: β= 0.055 (0.029, 0.082)<br>BMI↑ sTfR↑ female: β= 0.017 (0.001, 0.033)<br>BMI↑ CRP↑ | Inverse | Positive |
| Grant, 2007, New Zealand[18] | Cross-sectional, 416, 6-23 m | BMI>16.5 & 18.5<br>ID: NHANES standard: ≥2 abnormal parameters: SF, TS and EP. | BMI↑ ID↑ BMI>18.5/ID: RR= 4.34 (1.08, 10.67) relative to BMI<16.5 | Inverse | Positive |
| Higgins, 2020, Canada[19] | Cross-sectional, 1332, 5.1-<19.0 y | NW, OW, OB: BMI>3$^{rd}$, 85$^{th}$ & 97$^{th}$ centiles<br>SI, serum transferrin | BMIz↑ serum transferrin↑ β:BMIz/transferrin: 0.061 (0.037, 0.085)<br>BMIz↑ SI↓ BMIz/iron: β= -0.768 (-1.31, -0.229) (male), -0.870 (-1.41- -0.329) (female) | Inverse | Positive |
| Kassem, 2022, Israel[20] | Cross-sectional, 146, 10-12 y | OW, OB: WHO standard<br>SF, TS<br>ID: SF<15μg/dL | BAZ↑ prevalence of ID↑<br>BAZ↑ TS↓, SF↑ | Inverse | Positive |
| Manios, 2013, Greece[21] | Cross-sectional, 2492, 9-13 y | OW, OB (International Obesity Task Force)<br>ID:TS<16%<br>IDA: ID + Hb<120g/L | BMI↑ ID, IDA↑<br>OB/ID: AOR= 2.46 (1.52, 3.96) (male), AOR= 2.05 (1.19, 3.51) (female)<br>OB/IDA: AOR= 3.13 (1.01, 7.91) (male), AOR= 3.28 (1.15, 9.33) (female) | Inverse | Positive |

**Tan *et al.* Supplementary Material**

| | | | | | |
|---|---|---|---|---|---|
| Moschonis, 2012, Greece[22] | Cross-sectional, 1493, 9-13 y | OW, OB (International Obesity Task Force)<br>ID:TS<16%<br>IDA: ID + Hb<120g/L | BMI↑ ID↑<br>OW/ID: AOR= 2.13 (1.27, 3.60) (male)<br>OB/ID: AOR= 2.25 (1.14, 4.46) (female) | Inverse | Neutral |
| Nead, 2004, US[23] | Cross-sectional, 9698, 2-16 y | OW, OB: BMI>85$^{th}$ & 95$^{th}$ centiles<br>ID: ≥ 2 abnormal parameters: TS, EP and SF | BMI↑ ID ↑<br>ID/OW: AOR= 2.0 (1.2, 3.5)<br>ID/OB: AOR= 2.3 (1.4, 3.9) | Inverse | Positive |
| Ortiz Perez, 2020, Spain[24] | Cross-sectional, 405, 12-16 y | OW, OB (International Obesity Task Force)<br>SF, CRP, sTfR, SI<br>elevated CRP were excluded. | BAZ↑ SF, CRP, sTfR↑ r= 0.14, r= 0.43, r= 0.17, respectively | Direct | Neutral |
| Pompano, 2022, Chile[25] | Cross-sectional, 518, 16-17 y | OW, OB: WHO standard<br>SF | BAZ↑ SF↑ r= 0.12 | Direct | Positive |
| Shattnawi, 2018, Jordan[26] | Cross-sectional, 873, 14.6±1.0 y | OW: BMIz: CDC standard<br>PF, elevated CRP were excluded. | BMI↑ PF↑ | Direct | Positive |
| Suteerojntrakool, 2021, Thailand[27] | Cross-sectional, 336, 6-12 y | OW, OB: WHO standard<br>ID: ≥2 abnormal parameters: TS<16%, SF<15μg/mL, and sTfR>5mg/L<br>IDA: ID + anaemia | BAZ↑ SF↑ r= 0.214;<br>BAZ↑ sTfR↑ r= 0.207;<br>BAZ↑ TS↓ r= -0.132;<br>BAZ↑ TIBC↑ r= 0.209 | Direct | Neutral |
| Sypes, 2018, Canada[28] | Cross-sectional, 1607 children, 1-3 y | BMIz<br>ID: SF<12μg/L<br>CRP>10mg/L was excluded | BMI↑ ID, SF↑<br>BMIz/ID: AOR= 1.28 (1.10, 1.50)<br>BMIz/median SF: β= -0.062 (-0.093, -0.031) | Inverse | Positive |
| Thillan, 2021, Sri Lanka[29] | Case-control, 324, 8-9 y | OW, OB: WHO standard<br>SF | BMI↑ SF↑ in boys | Direct | Positive |

**Tan *et al.* Supplementary Material**

| | | | | | |
|---|---|---|---|---|---|
| Tussing-Humphreys, 2009, US[30] | Cross-sectional, 210, 12-17 y | Heavier weight: BMI≥85$^{th}$ centile<br>ID: ≥2 abnormal parameters: MCV<82fL for 12-14y and<85fL for 15-17y, free EP >70µg/dL and TS<16%. | BMI↑ ID↑ BMI>85$^{th}$/ID: AOR= 2.32 (1.14, 4.71)<br>BMI↑ log SI↓ β= -0.004 | Inverse | Positive |
| Yalcin, 2019, Turkey[31] | Case-control, 120, 6-10 y | OW, OB: BAZ>1 & 2 SD<br>Whole blood iron | NS | NS | Positive |
| **Undernutrition (n=15)** | | | | | |
| Alaofe, 2017, Benin[32] | Cross-sectional, 647, 6-59 m | Stunting<br>Anaemia: WHO standard<br>ID: SF adjusted by medical status at birth<br>IDA: anaemia + ID | HAZ↑ ID, IDA↑<br>Stunting/ID: AOR= 2.17 (1.17, 4.02)<br>Stunting/IDA: AOR= 2.16 (1.05, 4.46) | Direct | Positive |
| Al-Hussaini, 2022, Saudi Arabia[33] | Case-control, 7931, 6-16 y | Thinness (wasting): BAZ<-2 SD<br>SI, SF | NS | NS | Neutral |
| Andre, 2017, Brazil[34] | Cross-sectional, 457, 4-7 y | HAZ<br>SF | NS | NS | Positive |
| Chitekwe, 2022, Nepal[35] | Cross-sectional, 1709, 6-59 m | Stunting<br>ID: SF<12.0ug/L or StfR>8.3mg/L | HAZ↓ ID↑<br>Stunting/ID (Ferritin): AOR= 1.61 (1.18, 2.18); Stunting/ID (StfR): AOR= 1.69 (1.20, 2.38) | Direct | Positive |
| Ernawati, 2021, Indonesia[36] | Cross-sectional, 1008, 11-16 y | Stunting, severe stunting<br>SF | NS | NS | Positive |
| Ghosh, 2012, Nepal[37] | Cross-sectional, 192, 6-10 y | Stunting: HAZ<-1 SD<br>SF | HAZ↑ SF↑ β= 0.217 | Direct | Negative |

**Tan *et al.* Supplementary Material**

| | | | | | |
|---|---|---|---|---|---|
| Habib, 2016, Pakistan[38] | Cross-sectional, 7138, 6-59 m | Stunting, Underweight: WAZ<-2 SD<br>IDA: Hb<110g/L, SF<12μg/L<br>Hb adjusted for altitudes | HAZ↓ IDA↑ stunting/IDA: AOR= 1.42 (1.23, 1.63)<br>WAZ↓ IDA↑ underweight/IDA COR= 1.28 (1.15, 1.43) | Direct | Positive |
| Indriastuti Kurniawan, 2006, Indonesia[39] | Cross-sectional, 133, 10-12 y | Thinness: BAZ<-2 SD<br>Anaemia: Hb<120g/L<br>IDA: Anaemia + SF<12μg/L, sTfR>8.5mg/L, ZnPP>40μmol/mol heme. | **Comparing with female patients with anaemia:**<br>BAZ↓ IDA↑ thinness/IDA: AOR= 5.1 (1.34, 19.00) | Direct | Negative |
| Khatib, 2009, Jordan[40] | Cross-sectional, 560, 5.5-10 y | Stunting<br>SF | HAZ↓ SF↓ | Direct | Neutral |
| Kumari, 2022, India[41] | Case-control, 202, 1-5 y | WHZ, BAZ<br>SI | WHZ↑ SI↑<br>BMI↑ SI↑ r= 0.301 | Direct | Neutral |
| Matsungo, 2017, South Africa[42] | Cross-sectional, 750, 0-6 m | Stunting: LAZ<-2 SD<br>Anaemia: Hb<110g/L<br>ID: sTfR>8·3mg/L; IDA: ID + anaemia | LAZ↓ ID↑, IDA↑ | Direct | Positive |
| Orsango, 2021, Ethiopia[43] | Cross-sectional, 331, 2-5 y | HAZ, WHZ<br>IDA: Hb <110g/L, adjusted SF<12μg/L | HAZ ↑ IDA↓ HAZ/IDA: AOR= 0.74 (0.56, 0.98) | Direct | Positive |
| Park, 2012, South Korea[44] | Cross-sectional, 101, 11.1±2.0 m | HAZ, WAZ, WHZ<br>ID: SF<12ng/mL and/or TS<15% | NS | NS | Positive |
| Sethy, 2014, India[45] | Cross-sectional, 144, 2-5 y | Underweight: WAZ<-2SD<br>Stunting<br>SI | WHZ↓ SI↓ (mean level, but r= -0.175)<br>HAZ↓ SI↓ | Direct | Negative |



Tan X, *et al. BMJ Glob Health* 2024; 9:e015135. doi: 10.1136/bmjgh-2024-015135

**Tan *et al.* Supplementary Material**

| | | | | | |
|---|---|---|---|---|---|
| Shukla, 2023, India[46] | Case-control, 442, 6-60 m | SAM: MUAC<11.5cm SF | MUAC↓ SF↓ | Direct | Neutral |

[1]Stunting refers to HAZ<-2 SD unless noted

[2]OR shown as OR (95%CI) unless noted; β coefficient shown as β (95%CI) unless noted

Abbreviations:

AOR: adjusted odds ratio; BAZ: BMI-age z score; BIS: body iron store; BMI: body mass index; COR: crude odds ratio; CRP: C-reaction protein; HAZ: height-for-age z score; Hb: haemoglobin; fL: femtoliter; ID: iron deficiency; IDA: iron deficiency anaemia; EP: erythrocyte protoporphyrin, NW: normal weight; NS: not significant; MCV: mean corpuscular volume; OB: obese; OR: odds ratio; OW: overweight; PF: plasma ferritin; PR: prevalence ratio; SF: serum ferritin; SI: serum iron; sTfR: serum transferrin receptor; TfR: transferrin receptor; TS: transferrin saturation; WAZ: weight-for-age z score; WHO: World Health Organization; WHZ: weight-for-height z score; SAM: severe acute malnutrition; ZnPP: zinc protoporphyrin.



Tan X, *et al. BMJ Glob Health* 2024; 9:e015135. doi: 10.1136/bmjgh-2024-015135

**Tan *et al.* Supplementary Material**

**Table S6 Characteristics of the studies (n=28) assessing the association between zinc and weight status.**

| **First Author, Year, Country** | **Study Design, Participant (n), age** | **Malnutrition and Biochemical Indicators[1]** | **Significant findings[2]** | **Association** | **Quality Rating** |
|---|---|---|---|---|---|
| **Both (n=4)** | | | | | |
| Dehghani, 2011, Iran[47] | Cross-sectional, 902, 3-18 y | Wasting, stunting, BMI percentiles<br>Serum Zn | **Overnutrition**: NS<br>**Undernutrition**:<br>Serum Zn↓ prevalence of mild wasting, stunting ↑ | NS<br><br>Direct | Neutral |
| Habib, 2022, Iran[48] | Cross-sectional, 454, 2-18 y | BMI percentiles, HAZ<br>Serum Zn | **Overnutrition**: NS<br>**Undernutrition**:<br>HAZ ↓Serum Zn↓ | NS<br><br>Direct | Positive |
| Li, 2017, Colombia[4] | Cross-sectional, 4279, 1-17 y | HAZ, BAZ<br>Serum Zn | **Both over- and undernutrition:** NS | NS | Positive |
| Lu, 2023, China[49] | Cross-sectional, 64850, 6-18 y | Stunting: WS/T 456-2014<br>OW, OB: WS/T 586-2018<br>Serum Zn | **Overnutrition:**<br>BMI↑ ZD↓ ZD/OW: AOR= 0.881 (0.808, 0.960);<br>ZD/OB: AOR= 0.776 (0.702, 0.857)<br>**Undernutrition:**<br>HAZ↓ ZD↑ AOR= 1.443 (1.19, 1.75) | Direct | Positive |
| **Overnutrition (n=8)** | | | | | |
| Fan, 2017, US[50] | Cross-sectional, 5404, 6-19 y | OW, OB: BMI percentiles<br>Serum Zn (vs Q1) | Serum Zn↑ BMI↓<br>Serum Zn Q4/OW: AOR= 0.65 (0.47, 0.89)<br>Serum Zn Q3/OB: AOR= 0.62 (0.41, 0.92)<br>(Serum Zn-BMI) male: β= -0.161 (-0.315, -0.007), female: β= -0.184 (-0.343, -0.025) | Inverse | Positive |
| Ho, 2017, Australia[51] | Cross-sectional, 726, 8 y (n=436), 15 y (n=290) | OW, OB (BMI z scores)<br>Plasma Zn | NS | NS | Neutral |
| Perrone, 1998, Italy[52] | Case-control, 207 (143+164), 4-16 y | OB: BMI>$95^{th}$ centile<br>Serum Zn | BMI↑ Serum Zn↓ | Inverse | Neutral |

**Tan *et al.* Supplementary Material**

| | | | | | |
|---|---|---|---|---|---|
| Sharif, 2019, Iran[53] | Cross-sectional, 4261, 15-23 m | OW: BAZ≥1 SD Quartiles of Zn levels (vs Q1); | NS | NS | Positive |
| Thillan, 2021, Sri Lanka[29] | Case-control, 324, 8-9 y | OW, OB: WHO standard Serum Zn | BMI↑ Serum Zn↑ (male) | Direct | Positive |
| Yalcin, 2019, Turkey[31] | Case-control, 120 (40+80), 6-10 y | OW, OB: BAZ>1 & 2 SD Whole blood Zn | BMI↑ Whole blood Zn↑ | Direct | Neutral |
| Zhu, 2021, China[54] | Cross-sectional, 3241, 6-17 y | OW, OB: BMI (WS/T 586—2018) Quartiles of Zn levels (vs Q1); | Serum Zn ↑ BMI ↑ | Direct | Positive |
| Zou, 2022, China[55] | Cross-sectional, 2818, 6-17 y | OW, OB: BMI (WS/T 586—2018) Serum Zn | NS | NS | Positive |
| **Undernutrition (n=16)** | | | | | |
| Al-Hussaini, 2022, Saudi Arabia[33] | Case-control, 7931, 6-16 y | Thinness(wasting): BAZ<-2SD Serum Zn | NS | NS | Neutral |
| Engle-Stone, 2014, Cameroon[56] | Cross-sectional, 817, 12-59 m | HAZ, WHZ Plasma Zn | HAZ, WHZ↓ Plasma Zn↓ | Direct | Positive |
| Galetti, 2016, Benin[57] | Cross-sectional, 598, 1-10 y | HAZ Plasma Zn | Plasma Zn↑ HAZ↑ HAZ/Plasma Zn: β= 0.15 (SE= 0.06) | Direct | Positive |
| Gibson, 2007, Thailand[58] | Case-control, 230 (58+172), 6-13 y | Stunting Serum Zn | NS | NS | Positive |
| Goyena, 2021, Philippine[59] | Cross-sectional, 2892, 6-71 m | Stunting ZD: IZiNCG standard | HAZ↓ ZD↑ ZD/stunting: AOR= 1.36 (1.06, 1.76) | Direct | Positive |
| Kongsbak, 2006, Bangladesh[60] | Cross-sectional, 579, 3-7 y | Stunting Serum Zn | HAZ↓ Serum Zn↓ stunting was associated with 0.2μmol/L lower Serum Zn. | Direct | Positive |
| Kumari, 2022, | Case-control, 202, | WHZ, BAZ Serum Zn | BMI↑ Serum Zn↑ r= 0.201 WHZ↑ Serum Zn↑ | Direct | Neutral |

**Tan *et al.* Supplementary Material**

| | | | | | |
|---|---|---|---|---|---|
| India[41] | 1-5 y | | | | |
| Marashinghe, 2015, Sri Lanka[61] | Cross-sectional, 340, 2-5 y | Underweight: WAZ<-2 SD, stunting, wasting/thinness: WHZ<-2 SD<br>Serum Zn | HAZ↑ Serum Zn↑ severe stunting vs. normal<br>WAZ↑ Serum Zn↑ severe underweight vs. normal | Direct | Neutral |
| Nasiri-Babadi, 2021, Iran[62] | Cross-sectional, 425, 5-7 y | Wasting (BAZ<-1)<br>Serum Zn, tertiles of Zn levels (vs T1) | Serum Zn↑ weight, BAZ↑<br>Wasting/Zn T3: AOR= 0.53 (0.31, 0.91) | Direct | Positive |
| Naupal-Forcadilla, 2017, Philippines[63] | Cross-sectional, 149, 2-3 y | HAZ<br>ZD: IZiNCG standard | HAZ↑ ZD↓ HAZ/ZD: AOR= 0.86 (0.78, 0.96)<br>ZD was less likely to occur among children with normal HAZ | Direct | Positive |
| Okafor, 2021, Nigria[64] | Cross-sectional, 380, 6-12 y | Stunting<br>Serum Zn<br>ZD: serum Zn<80µg/dL | NS | NS | Positive |
| Park, 2012, South Korea[44] | Cross-sectional, 101, 6-24 m | HAZ, WAZ, WHZ: <-1 SD<br>Hypozincaemia: serum Zn<70µg/dL | NS | NS | Positive |
| Sethy, 2014, India[45] | Cross-sectional, 144, 2-5 y | Stunting, wasting: WHZ<-2 SD<br>Serum Zn | HAZ↓ Serum Zn↓ (mean level, but r= -0.193);<br>WHZ↓ Serum Zn↓ (mean level, but r= -0.278) | Direct | Negative |
| Tessema, 2019, Ethiopia[65] | Cross-sectional, 1776, 6-59 m | HAZ, WAZ<br>Serum Zn, adjusted by CRP, AGP | NS | NS | Neutral |
| Van Nhien, 2009, Vietnam[66] | Cross-sectional, 245, 11-17 y | BMI<17<br>Serum Zn | NS | NS | Neutral |
| Yazbeck, 2016, Lebanon[67] | Case-control, 161(78+83), 1-10 y | FTT: WAZ<$5^{th}$ centile;<br>Short stature: HAZ<-2 SD or <$2.5^{th}$ centile<br>ZD: Plasma Zn<65µg/dL | NS | NS | Neutral |

[1]Stunting refers to HAZ<-2 SD, severe stunting refers to HAZ<-3SD unless specific noted.
[2]OR were shown as OR (95%CI) unless specific noted; β coefficient were shown as β (95%CI) unless specific noted. NS: not significant.
AGP: α-acid glycoprotein; AOR: adjusted odds ratio; AOR: adjusted odds ratio; BAZ: BMI-age z score; BMI: body mass index; CRP: C-reaction protein; FTT: Failure to thrive; HAZ: height-for-age z score; IZiNCG: International Zinc Nutrition Consultative Group; MUAC: mid-upper arm circumference; OB: obese; OR: odds ratio; OW: overweight; SE: standard error; WAZ: weight-for-age z score; WHZ: weight-for-height z score; Zn: zinc; zinc deficiency: ZD.

**Tan *et al.* Supplementary Material**

**Table S7 Characteristics of the studies (n=27) assessing the association between vitamin A and weight status.**

| First Author, Year, Country | Study Design, Participant (n), age | Malnutrition and Biochemical Indicators[1] | Significant findings[2] | Association | Quality Rating |
|---|---|---|---|---|---|
| **Both (n=5)** | | | | | |
| Dallazen, 2023, Brazil[68] | Cross-sectional, 1503, 12-59 m | HAZ, BAZ<br>VAD: plasma retinol<0.7μmol/L | **Overnutrition**:<br>BAZ↑ plasma retinol↓<br>**Undernutrition**:<br>HAZ↓ VAD↑ APR= 4.75 (2.10, 10.73) plasma retinol↓ | Inverse<br><br>Direct | Positive |
| Disalvo, 2019, Argentina[69] | Cross-sectional, 624, 1-6 y | WAZ, BMIz<br>Serum retinol | **Overnutrition**: BMIz↑ Serum VA↑<br>**Undernutrition**:<br>WAZ↓ Serum retinol↓ | Direct | Neutral |
| Li, 2017, Colombia[4] | Cross-sectional, 3844, 1-17 y | HAZ, BAZ<br>Serum VA | **Both under- and overnutrition**:<br>BAZ↑ Serum VA↑ | Direct | Positive |
| Maslova, 2009, Colombia[5] | Cross-sectional, 2811, 5-12 y | BAZ<br>Plasma retinol | **Both under- and overnutrition**:<br>Plasma retinol↑ BAZ↑ | Direct | Positive |
| Tan, 2023, Malaysia[8] | Cross-sectional, 776, 7-11 y | BAZ, HAZ, WAZ<br>VAD: Both plasma retinol and RBP<0.7μmol/L | **Overnutrition**:<br>BAZ↑ retinol, RBP↑; WAZ↑ retinol, RBP↑<br>**Undernutrition**:<br>Serum VA↓ HAZ↓ VAD/stunting: COR= 2.9 (1.8, 4.7) | Direct | Positive |
| **Overnutrition (n=12)** | | | | | |
| Cobayashi, 2013, Brazil[1] | Cross-sectional, 582, 0-10 y | OW: BAZ>1<br>VAD: plasma VA<20μg/dL | OW↑ VAD↑ PR= 1.97 (1.13, 3.41) for ≥5y | Inverse | Positive |
| de Souza Valente da Silva, | Cross-sectional, 471, 7-17 y | OW, OB: BMI>85th & 95th centiles<br>Low serum carotenoids:<40 μg/dL | BMI↑ serum carotenoids↓ r= -0.192<br>BMI/low serum carotenoids: β= 0.92, AOR= 2.51 (1.43, 4.39) | Inverse | Positive |

**Tan *et al.* Supplementary Material**

| | | | | | |
|---|---|---|---|---|---|
| 2007,<br>Brazil[70] | | | | | |
| Gunanti,<br>2014,<br>US[71] | Cross-sectional,<br>1154,<br>8-15 y | OW, OB: BMI>85$^{th}$ & 95$^{th}$ centiles<br>Serum retinol and quartiles | BMI↑ serum retinol↑ β= 5.56 (3.36, 7.75)<br>OW/retinol quartile: AOR= 2.01(1.26, 3.22)<br>OB/retinol quartile: AOR= 2.90(1.65, 5.09) | Direct | Positive |
| Hu,<br>2001,<br>China[72] | Cross-sectional,<br>793,<br>0-19 y | BMI<br>Serum VA | BMI↑ VA↑ r= 0.26, β= 4.32±0.93<br>Weight↑ VA↑ r= 0.37, β= 10.39±1.85 | Direct | Positive |
| Ortega-Senovilla,<br>2019,<br>Spain[73] | Case-control,<br>141 (70+71),<br>6-8 y | OB (Cole et al, 2000)<br>Plasma all-trans-retinol | BMI↑ plasma retinol↑ | Direct | Neutral |
| Paes-Silva,<br>2018,<br>Brazil[74] | Cross-sectional,<br>411,<br>12-19 y | OW: BAZ>1;<br>Low β-carotene: Serum β-carotene <0.9μmol/L | BAZ↑ β-carotene↓<br>Low β-carotene/OW, PR= 1.46 (1.2, 1.8) (male)<br>Low β-carotene/NW, PR= 1.19 (1.0, 1.4) (female) | Inverse | Neutral |
| Sharif,<br>2019,<br>Iran[53] | Cross-sectional,<br>4261,<br>15-23 m | OW: BAZ>1<br>Quartiles of retinol levels (vs Q1) | NS | NS | Positive |
| Thillan,<br>2021,<br>Sri Lanka[29] | Case-control,<br>324,<br>8-9 y | OW; OB: WHO standard<br>Serum VA | NS | NS | Positive |
| Tian,<br>2022,<br>China[75] | Cross-sectional,<br>3025,<br>7-17 y | General OB: WS/T 586-2018<br>Central OB: WC≥90$^{th}$ centile<br>Quartiles of Serum VA levels (vs Q1) | serum VA↑ general OB↑ Q4/general OB: AOR= 4.10 (2.88, 5.85)<br>serum VA↑ central OB↑ Q4/central OB: AOR= 4.14 (3.07, 5.56) | Direct | Positive |
| Wei,<br>2016,<br>China[76] | Cross-sectional,<br>1928,<br>7-11 y | OB: BMI>95$^{th}$ centile<br>VAD: Serum VA <20μg/dL | BMI↑ VAD/OB: AOR= 2.37 (1.59, 3.55)<br>BMI↑ Serum VA↓ | Inverse | Positive |
| Yang,<br>2015,<br>China[77] | Cross-sectional,<br>3457,<br>7-17 y | OW, OB: BMI>85$^{th}$ & 95$^{th}$ centiles<br>VAD: serum retinol<30μg/dL | BMI↑ serum retinol↑<br>VAD/NW: AOR= 1.34 (1.10, 1.63), β= 0.29 | Direct | Positive |
| Zou,<br>2022, | Cross-sectional,<br>2818 | OW; OB: WS/T 586-2018<br>VAI: Serum VA 20-30μg/dL; | serum VA↑ BMI↑<br>sufficient VA/OW: AOR= 1.55 (1.19, 2.02) | Direct | Positive |

**Tan *et al.* Supplementary Material**

| | | | | | |
|---|---|---|---|---|---|
| China[55] | 6-17 y | VAD: Serum VA<20μg/dL | | | |
| **Undernutrition (n=10)** | | | | | |
| Adamu, 2016, Nigeria[78] | Case-control, 550 (275+275), 6 m-5 y | WHZ<br>Serum retinol | WHZ↓ Serum VA↓ | Direct | Neutral |
| Ahmed, 2006, Bangladesh[79] | Cross-sectional, 381, 11-16 y | BMI<br>Serum VA | BMI↓ Serum VA↓ r= 0.33, β= 0.26 | Direct | Positive |
| Alaofe, 2017, Benin[32] | Cross-sectional, 647 children, 6-59 m | Stunting<br>VAD: serum retinol<20μg/dL, corrected by infection | NS | NS | Positive |
| Ernawati, 2021, Indonesia[36] | Cross-sectional, 1008, 11-16 y | Stunting; severe stunting<br>Serum retinol | HAZ↓ serum retinol↓ | Direct | Positive |
| Khatib, 2009, Jordan[40] | Cross-sectional, 560, 5.5-10 y | BMI; stunting<br>Serum VA | BMI↓ serum VA↓ r= 0.114 | Direct | Neutral |
| Kurugol, 2000, Turkey[80] | Cross-sectional, 160, 6-59 m | Stunting<br>Serum retinol | HAZ↓ Serum retinol↓ | Direct | Neutral |
| Marashinghe, 2015, Sri Lanka[61] | Cross-sectional, 340, 2-5 y | Underweight: WAZ<-2; stunting; wasting/thinness: WHZ<-2<br>Serum VA | NS | NS | Neutral |
| Oso, 2003, Nigeria[81] | Cross-sectional, 213, 6 m-6 y | Stunting<br>Serum retinol | HAZ↓ serum retinol↓ | Direct | Negative |
| Samba, 2006, Congo[82] | Cross-sectional, 5722, 0-71 m | WHZ <-2 SD<br>Serum retinol | WHZ↓ Serum retinol↓ | Direct | Neutral |



Tan X, *et al. BMJ Glob Health* 2024; 9:e015135. doi: 10.1136/bmjgh-2024-015135

**Tan *et al.* Supplementary Material**

| | | | | | |
|---|---|---|---|---|---|
| Ssentongo, 2020, Uganda[83] | Cross-sectional, 4765, 6–59 m | Stunting; severe stunting<br>VAD: RBP<0.83μmol/L, adjusted by CRP | RBP↓ HAZ↓<br>VAD/stunting: AOR= 1.43 (1.08, 1.89)<br>VAD/severe stunting: AOR= 1.64 (1.14, 2.35) | Direct | Positive |

[1]Stunting refers to HAZ<-2 SD, severe stunting refers to HAZ<-3SD unless specific noted.
[2]OR were shown as OR (95%CI) unless specific noted; β coefficient were shown as β (95%CI) unless specific noted.

AOR: adjusted odds ratio; BAZ: BMI-age z score; BMI: body mass index; CRP: C-reaction protein; HAZ: height-for-age z score; NS: not significant; NW: normal weight; OB: obese; OR: odds ratio; OW: overweight; RBP: retinol binding protein; VAD: vitamin A deficiency; WAZ: weight-for-age z score; WHZ: weight-for-height z score.

**Tan *et al.* Supplementary Material**

**Table S8 Risk of bias assessment for included studies.**

| First Author, Year | Micronutrient | Nutrition | Q1 | Q2 | Q3 | Q4 | Q5 | Q6 | Q7 | Q8 | Q9 | Q10 | Overall Rating |
|---|---|---|---|---|---|---|---|---|---|---|---|---|---|
| Abd-El-Wahed, 2014 | Iron | Overnutrition | YES | YES | NO | NO | YES | YES | YES | NO | YES | NO | NEUTRAL |
| Adamu, 2016 | VA | Undernutrition | YES | YES | NO | NO | YES | YES | YES | NO | NO | YES | NEUTRAL |
| Ahmed, 2006 | VA | Undernutrition | YES | YES | YES | YES | YES | YES | YES | YES | YES | NO | POSITIVE |
| Alaofe, 2017 | Iron, VA | Undernutrition | YES | YES | YES | YES | YES | YES | YES | YES | YES | YES | POSITIVE |
| Al-Hussaini, 2022 | Iron, zinc | Undernutrition | YES | YES | YES | NO | YES | YES | NO | NO | YES | YES | NEUTRAL |
| Andre, 2017 | Iron | Undernutrition | YES | YES | YES | NO | YES | YES | YES | YES | NO | NO | POSITIVE |
| Brotanek, 2007 | Iron | Overnutrition | YES | YES | YES | NO | YES | YES | YES | YES | YES | YES | POSITIVE |
| Cabanas Pujadas, 2022 | Iron | Overnutrition | YES | YES | YES | NO | YES | YES | YES | YES | YES | YES | POSITIVE |
| Cepeda-Lopez, 2011 | Iron | Overnutrition | YES | YES | YES | NO | YES | YES | YES | YES | YES | YES | POSITIVE |
| Cheng, 2013 | Iron | Overnutrition | YES | YES | YES | NO | YES | YES | YES | YES | YES | YES | POSITIVE |
| Chitekwe, 2022 | Iron | Undernutrition | YES | YES | YES | YES | YES | YES | YES | YES | YES | YES | POSITIVE |
| Cobayashi, 2013 | Iron, VA | Both | YES | YES | YES | YES | YES | YES | YES | YES | YES | YES | POSITIVE |
| Dallazen, 2023 | VA | Both | YES | YES | YES | YES | YES | YES | YES | YES | YES | YES | POSITIVE |
| de Araujo, 2022 | Iron | Overnutrition | YES | YES | YES | YES | YES | YES | NO | NO | YES | YES | NEUTRAL |
| de Souza Valente da Silva, 2007 | VA | Overnutrition | YES | YES | YES | YES | YES | YES | YES | YES | NO | NO | POSITIVE |
| Dehghani, 2011 | Zinc | Both | YES | YES | NO | NO | YES | YES | YES | NO | NO | YES | NEUTRAL |
| Disalvo, 2019 | VA | Both | YES | YES | YES | NO | YES | NO | YES | YES | YES | YES | NEUTRAL |
| Eftekhari, 2009 | Iron | Both | YES | YES | NO | NO | YES | YES | YES | YES | NO | YES | NEUTRAL |
| El Khoury, 2020 | Iron | Overnutrition | YES | YES | NO | NO | YES | YES | NO | NO | YES | NO | NEUTRAL |
| Engle-Stone, 2014 | Zinc | Undernutrition | YES | YES | YES | YES | YES | YES | YES | YES | NO | YES | POSITIVE |
| Ernawati, 2021 | Iron, VA | Undernutrition | YES | YES | YES | NO | YES | YES | YES | YES | YES | YES | POSITIVE |
| Fan, 2017 | Zinc | Overnutrition | YES | YES | YES | NO | YES | YES | YES | YES | YES | YES | POSITIVE |
| Ferrari, 2015 | Iron | Overnutrition | YES | YES | YES | NO | YES | YES | YES | YES | YES | YES | POSITIVE |
| Galetti, 2016 | Zinc | Undernutrition | YES | YES | YES | NO | YES | YES | YES | YES | NO | YES | POSITIVE |
| Ghosh, 2012 | Iron | Undernutrition | YES | YES | NO | NO | YES | YES | NO | NO | NO | NO | NEGATIVE |
| Gibson, 2007 | Zinc | Undernutrition | YES | YES | YES | YES | YES | YES | YES | NO | NO | YES | POSITIVE |

**Tan *et al.* Supplementary Material**

| | | | | | | | | | | | | | |
|---|---|---|---|---|---|---|---|---|---|---|---|---|---|
| Goyena, 2021 | Zinc | Undernutrition | YES | YES | YES | YES | YES | YES | YES | YES | YES | YES | POSITIVE |
| Grant, 2007 | Iron | Overnutrition | YES | YES | YES | YES | YES | YES | YES | YES | NO | YES | POSITIVE |
| Gunanti, 2014 | VA | Overnutrition | YES | YES | YES | YES | YES | YES | YES | YES | YES | YES | POSITIVE |
| Habib, 2016 | Iron | Undernutrition | YES | YES | YES | YES | YES | YES | YES | YES | YES | YES | POSITIVE |
| Habib, 2022 | Zinc | Both | YES | YES | YES | NO | YES | YES | YES | NO | YES | YES | POSITIVE |
| Higgins, 2020 | Iron | Overnutrition | YES | YES | YES | NO | YES | YES | YES | YES | YES | YES | POSITIVE |
| Ho, 2017 | Zinc | Overnutrition | YES | Unclear | NO | NO | YES | YES | YES | NO | YES | YES | NEUTRAL |
| Hu, 2001 | VA | Overnutrition | YES | YES | YES | NO | YES | YES | YES | YES | YES | NO | POSITIVE |
| Indriastuti Kurniawan, 2006 | Iron | Undernutrition | YES | YES | NO | NO | YES | YES | NO | NO | NO | NO | NEGATIVE |
| Kassem, 2022 | Iron | Overnutrition | YES | YES | YES | YES | YES | YES | YES | NO | YES | YES | POSITIVE |
| Khatib, 2009 | Iron, VA | Undernutrition | YES | YES | NO | YES | YES | YES | YES | NO | YES | YES | NEUTRAL |
| Kongsbak,  2006 | Zinc | Undernutrition | YES | YES | YES | YES | YES | YES | YES | YES | YES | YES | POSITIVE |
| Kumari, 2022 | Iron, zinc | Undernutrition | YES | NO | YES | NO | YES | YES | YES | NO | YES | YES | NEUTRAL |
| Kurugol, 2000 | VA | Undernutrition | YES | YES | NO | NO | YES | YES | YES | NO | NO | YES | NEUTRAL |
| Li, 2017 | Iron, zinc, VA | Both | YES | YES | YES | NO | YES | YES | YES | YES | YES | YES | POSITIVE |
| Lu, 2023 | Zinc | Both | YES | YES | YES | NO | YES | YES | YES | YES | YES | YES | POSITIVE |
| Manios,2013 | Iron | Overnutrition | YES | YES | YES | YES | YES | YES | YES | YES | YES | YES | POSITIVE |
| Marashinghe, 2015 | Zinc, VA | Undernutrition | YES | YES | YES | NO | YES | NO | YES | NO | YES | YES | NEUTRAL |
| Maslova, 2009 | Iron, VA | Both | YES | YES | YES | YES | YES | YES | YES | YES | YES | YES | POSITIVE |
| Matsungo, 2017 | Iron | Undernutrition | YES | YES | YES | NO | YES | YES | YES | YES | YES | YES | POSITIVE |
| Moschonis, 2012 | Iron | Overnutrition | YES | YES | YES | NO | YES | YES | NO | YES | YES | YES | NEUTRAL |
| Nasiri-babadi, 2021 | Zinc | Undernutrition | YES | YES | YES | NO | YES | YES | YES | YES | YES | YES | POSITIVE |
| Naupal-Forcadilla, 2017 | Zinc | Undernutrition | YES | YES | YES | NO | YES | YES | YES | YES | NO | YES | POSITIVE |
| Nead, 2004 | Iron | Overnutrition | YES | YES | YES | NO | YES | YES | YES | YES | YES | NO | POSITIVE |
| Okafor, 2021 | Zinc | Undernutrition | YES | YES | YES | NO | YES | YES | YES | NO | YES | YES | POSITIVE |
| Onabanjo, 2014 | Iron | Both | YES | NO | NO | YES | YES | YES | NO | NO | YES | YES | NEUTRAL |
| Orsango, 2021 | Iron | Undernutrition | YES | YES | YES | NO | YES | YES | YES | YES | YES | YES | POSITIVE |
| Ortega-Senovilla, 2019 | VA | Overnutrition | YES | YES | YES | NO | YES | YES | NO | NO | YES | YES | NEUTRAL |
| Ortiz Perez, 2020 | Iron | Overnutrition | YES | YES | NO | YES | YES | YES | YES | NO | YES | YES | NEUTRAL |



Tan X, *et al. BMJ Glob Health* 2024; 9:e015135. doi: 10.1136/bmjgh-2024-015135

**Tan *et al.* Supplementary Material**

| | | | | | | | | | | | | | |
|---|---|---|---|---|---|---|---|---|---|---|---|---|---|
| Oso, 2003 | VA | Undernutrition | YES | YES | NO | YES | YES | YES | YES | NO | NO | NO | NEUTRAL |
| Paes-Silva, 2018 | VA | Overnutrition | YES | YES | NO | NO | YES | YES | YES | YES | YES | YES | NEUTRAL |
| Park, 2012 | Iron, zinc | Undernutrition | YES | YES | YES | NO | YES | YES | YES | YES | YES | YES | POSITIVE |
| Perng, 2013 | Iron | Both | YES | YES | YES | NO | YES | YES | YES | NO | YES | YES | POSITIVE |
| Perrone, 1998 | Zinc | Overnutrition | YES | NO | NO | NO | YES | YES | YES | NO | YES | NO | NEUTRAL |
| Pompano, 2022 | Iron | Overnutrition | YES | YES | YES | YES | YES | YES | YES | NO | YES | YES | POSITIVE |
| Samba, 2006 | VA | Undernutrition | YES | YES | NO | NO | YES | YES | YES | NO | NO | YES | NEUTRAL |
| Sethy, 2014 | Iron, zinc | Undernutrition | YES | NO | NO | NO | YES | YES | YES | NO | NO | NO | NEGATIVE |
| Sharif, 2019 | zinc, VA | Overnutrition | YES | YES | YES | YES | YES | YES | YES | YES | YES | YES | POSITIVE |
| Shattnawi, 2018 | Iron | Overnutrition | YES | YES | YES | YES | YES | YES | YES | YES | YES | YES | POSITIVE |
| Shukla, 2023 | Iron | Undernutrition | YES | NO | YES | NO | YES | YES | NO | NO | NO | YES | NEUTRAL |
| Ssentongo, 2020 | VA | Undernutrition | YES | YES | YES | NO | YES | YES | YES | YES | YES | YES | POSITIVE |
| Suteerojntrakoo, 2021 | Iron | Overnutrition | YES | YES | YES | YES | YES | YES | NO | YES | YES | YES | NEUTRAL |
| Sypes, 2018 | Iron | Overnutrition | YES | YES | YES | YES | YES | YES | YES | YES | YES | YES | POSITIVE |
| Tan, 2023 | Iron | Both | YES | YES | YES | NO | YES | YES | YES | YES | YES | YES | POSITIVE |
| Tessema, 2019 | Zinc | Undernutrition | YES | YES | NO | YES | YES | NO | YES | YES | YES | YES | NEUTRAL |
| Thillan, 2021 | Iron, zinc, VA | Overnutrition | YES | YES | YES | NO | YES | YES | YES | NO | YES | YES | POSITIVE |
| Tian, 2022 | VA | Overnutrition | YES | YES | YES | NO | YES | YES | YES | YES | YES | YES | POSITIVE |
| Tussing-Humphreys, 2009 | Iron | Overnutrition | YES | YES | YES | NO | YES | YES | YES | YES | YES | YES | POSITIVE |
| Van Nhien, 2009 | Zinc | Undernutrition | YES | YES | NO | YES | YES | YES | YES | NO | YES | YES | NEUTRAL |
| Wei, 2016 | VA | Overnutrition | YES | YES | YES | YES | YES | YES | YES | YES | YES | YES | POSITIVE |
| Yalcin, 2019 | Iron, zinc | Overnutrition | YES | YES | YES | NO | YES | YES | NO | NO | YES | YES | NEUTRAL |
| Yang, 2015 | VA | Overnutrition | YES | YES | YES | NO | YES | YES | YES | NO | YES | YES | POSITIVE |
| Yazbeck, 2016 | Zinc | Undernutrition | YES | YES | NO | NO | YES | YES | YES | NO | YES | YES | NEUTRAL |
| Zhu, 2019 | Iron | Both | YES | YES | YES | NO | YES | YES | NO | YES | YES | UES | NEUTRAL |
| Zhu, 2021 | Zinc | Overnutrition | YES | YES | YES | YES | YES | YES | YES | YES | YES | YES | POSITIVE |
| Zimmermann, 2008 | Iron | Both | YES | Unclear | YES | NO | YES | YES | YES | YES | YES | YES | NEUTRAL |
| Zou, 2022 | Zinc, VA | Overnutrition | YES | YES | YES | NO | YES | YES | YES | YES | YES | YES | POSITIVE |



Tan X, *et al. BMJ Glob Health* 2024; 9:e015135. doi: 10.1136/bmjgh-2024-015135

**Tan *et al.* Supplementary Material**

**Figure S1** Subgroup analysis of associations between iron deficiency and overnutrition stratified by gender. The vertical line represents no effect (OR=1.00) and the dashed line the overall effect (OR=1.51).

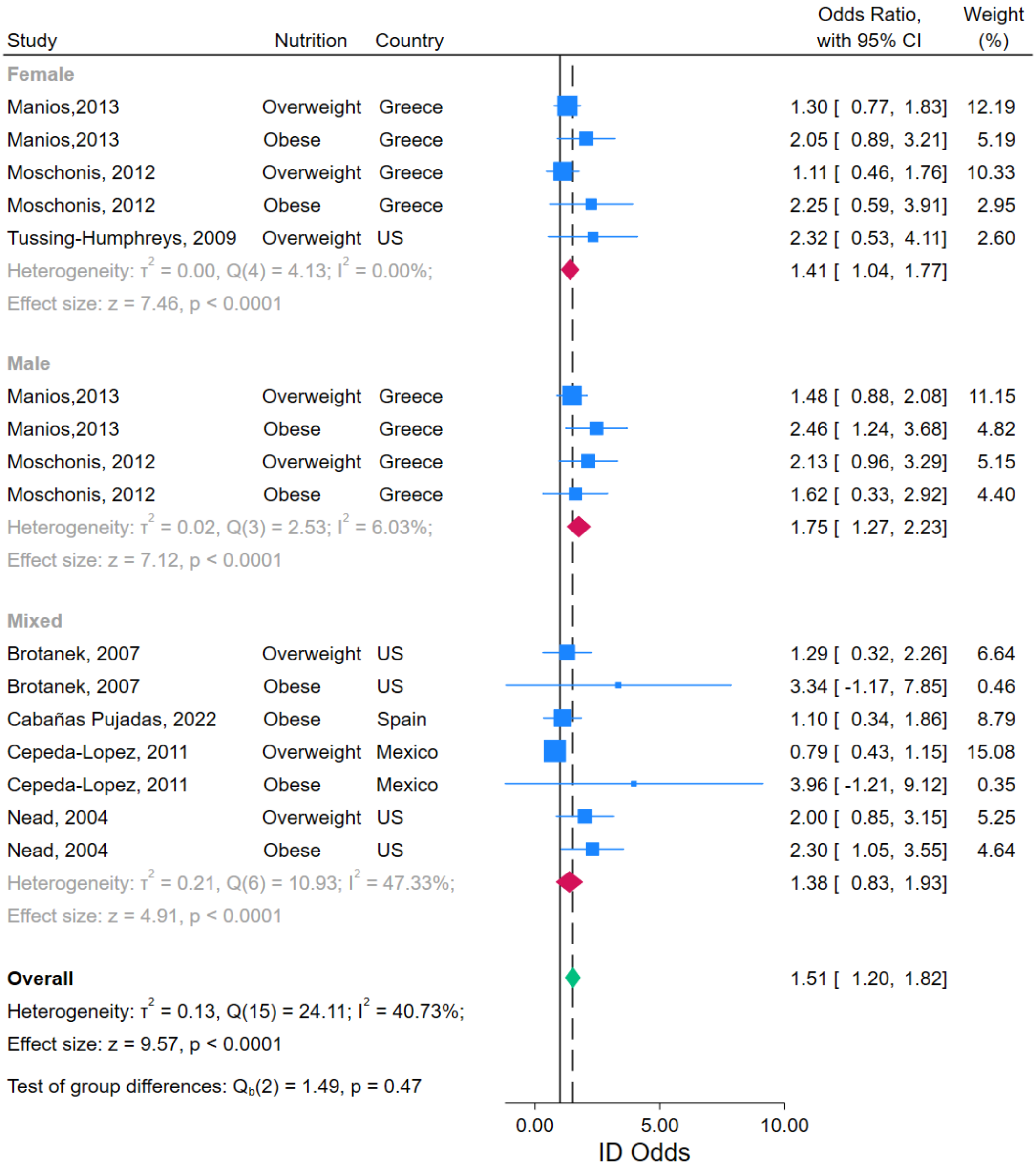

**Tan *et al.* Supplementary Material**

**Figure S2** Funnel plots for nutrition status and gender. **A.** nutrition status and **B.** gender. The red line represents the odds ratio for each group.

**A.**

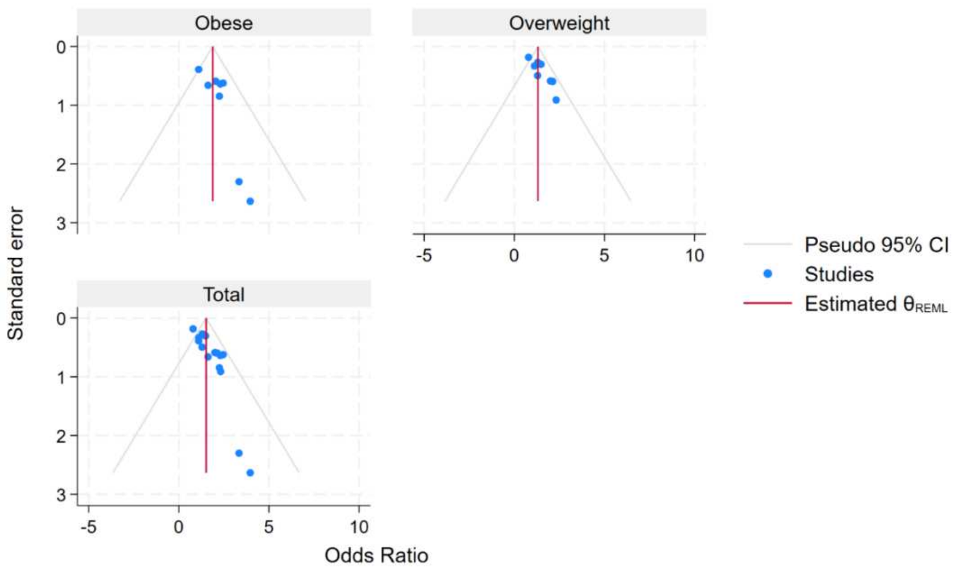


**B.**

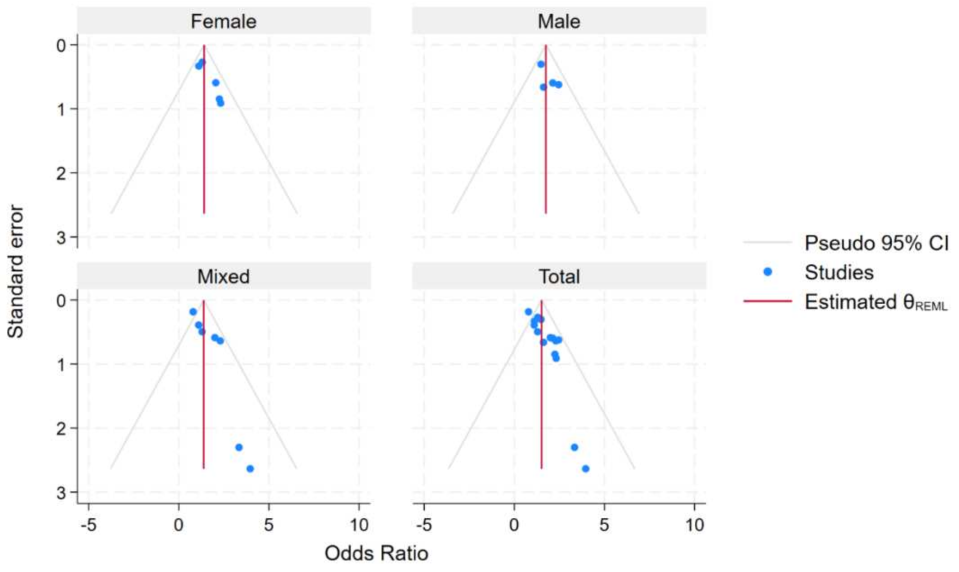

**Tan *et al.* Supplementary Material**

**Figure S3** Leave-one-out sensitivity test for overall and subgroup effect sizes. The red line represents the effect size before omitting any study. **A.** Overall, **B.** obese, **C.** overweight.

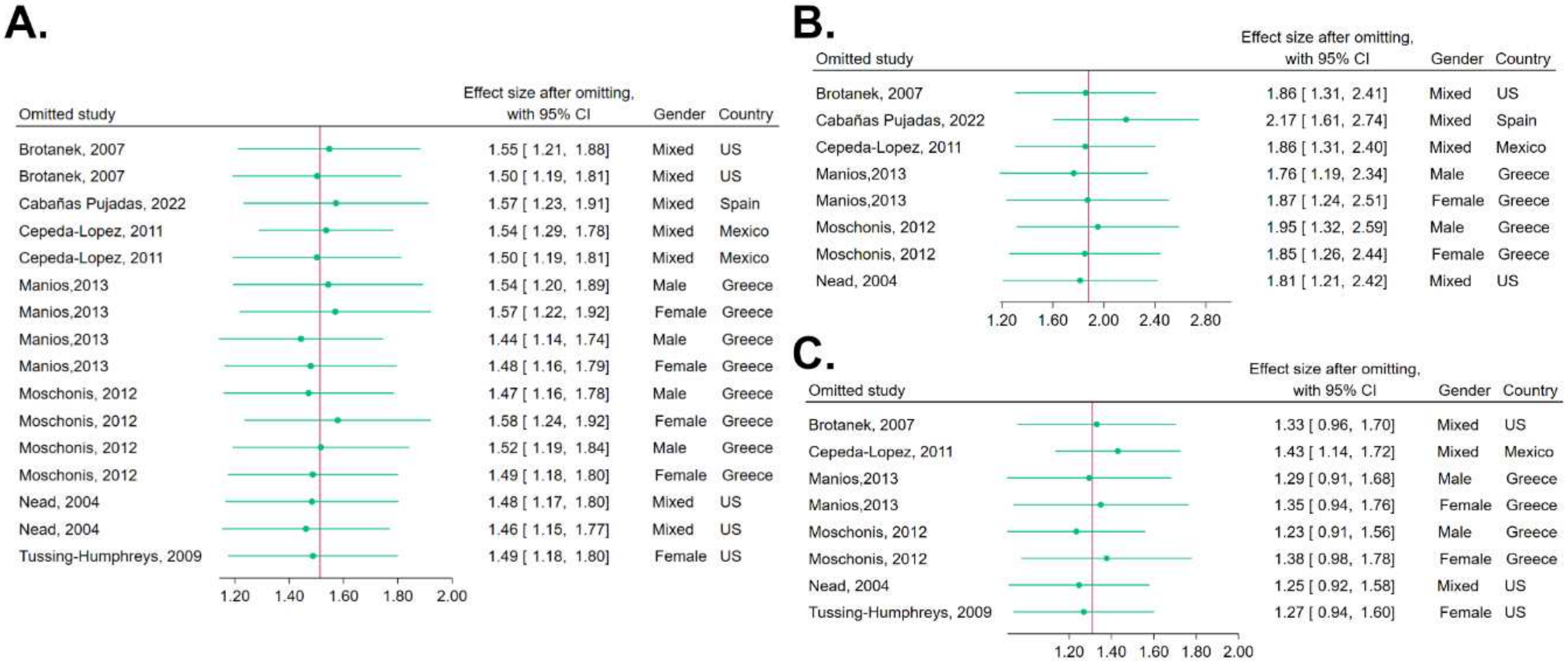

**Tan *et al.* Supplementary Material**

## References


1. Cobayashi F, Augusto RA, Lourenco BH, et al. Factors associated with stunting and overweight in Amazonian children: a population-based, cross-sectional study. *Public Health Nutr* 2014;17(3):551-60. doi: 10.1017/S1368980013000190
2. El Khoury R, Sleilaty G, Gannage-Yared MH. Prevalence of Iron deficiency in Lebanese schoolchildren. *Eur J Clin Nutr* 2020;74(8):1157-63. doi: 10.1038/s41430-020-0590-y
3. Eftekhari M, Mozaffari-Khosravi H, Shidfar F. The relationship between BMI and iron status in iron-deficient adolescent Iranian girls. *Public Health Nutr* 2009;12(12):2377-81. doi: 10.1017/S1368980009005187
4. Li W, Herran OF, Villamor E. Trends in Iron, Zinc, and Vitamin A Status Biomarkers Among Colombian Children: Results From 2 Nationally Representative Surveys. *Food Nutr Bull* 2017;38(2):146-57. doi: 10.1177/0379572117700976
5. Maslova E, Mora-Plazas M, Forero Y, et al. Are Vitamin A and Iron Deficiencies Re-Emerging in Urban Latin America? A Survey of Schoolchildren in Bogota, Colombia. *Food Nutr Bull* 2009;30(2):103-11. doi: 10.1177/156482650903000201
6. Onabanjo OO, Balogun OL. Anthropometric and Iron Status of Adolescents From Selected Secondary Schools in Ogun State, Nigeria. *ICAN: Infant, Child, & Adolescent Nutrition* 2014;6(2):109-18. doi: 10.1177/1941406414520703
7. Perng W, Mora-Plazas M, Marin C, Villamor E. Iron status and linear growth: a prospective study in school-age children. *Eur J Clin Nutr* 2013;67(6):646-51. doi: 10.1038/ejcn.2013.56
8. Tan PY, Mohd Johari SN, Teng KT, et al. High prevalence of malnutrition and vitamin A deficiency among schoolchildren of rural areas in Malaysia using a multi-school assessment approach. *Br J Nutr* 2023;129(3):454-67. doi: 10.1017/S0007114522001398
9. Zhu Y, He B, Xiao Y, Chen Y. Iron metabolism and its association with dyslipidemia risk in children and adolescents: a cross-sectional study. *Lipids Health Dis* 2019;18(1):50. doi: 10.1186/s12944-019-0985-8
10. Zimmermann MB, Zeder C, Muthayya S, et al. Adiposity in women and children from transition countries predicts decreased iron absorption, iron deficiency and a reduced response to iron fortification. *Int J Obes (Lond)* 2008;32(7):1098-104. doi: 10.1038/ijo.2008.43
11. Abd-El Wahed MA, Mohamed MH, Ibrahim SS, El-Naggar WA. Iron profile and dietary pattern of primary school obese Egyptian children. *J Egypt Public Health Assoc* 2014;89(2):53-9. doi: 10.1097/01.EPX.0000451827.84315.5c
12. Brotanek JM, Gosz J, Weitzman M, Flores G. Iron deficiency in early childhood in the United States: risk factors and racial/ethnic disparities. *Pediatrics* 2007;120(3):568-75. doi: 10.1542/peds.2007-0572
13. Cabañas Pujadas G, Ortiz-Marrón H, Ortiz-Pinto MA, et al. Changes in obesity and iron deficiency between 4 and 9 years of age. Longitudinal study of childhood obesity (ELOIN). *Int J Obes (Lond)* 2022;46(11):1992-99. doi: 10.1038/s41366-022-01196-y
14. Cepeda-Lopez AC, Osendarp SJ, Melse-Boonstra A, et al. Sharply higher rates of iron deficiency in obese Mexican women and children are predicted by obesity-related inflammation rather than by differences in dietary iron intake. *Am J Clin Nutr* 2011;93(5):975-83. doi: 10.3945/ajcn.110.005439
15. Cheng HL, Bryant CE, Rooney KB, et al. Iron, hepcidin and inflammatory status of young healthy overweight and obese women in Australia. *PLoS One* 2013;8(7):e68675. doi: 10.1371/journal.pone.0068675
16. de Araújo LKAR, Faria JCP, Sarni ROS. Iron deficiency anemia in infants in Sousa (PB), Brazil: an association with nutritional status. *Revista da Associacao Medica Brasileira* 2022;68(12):1698-704. doi: 10.1590/1806-9282.20220761
17. Ferrari M, Cuenca-Garcia M, Valtuena J, et al. Inflammation profile in overweight/obese adolescents in Europe: an analysis in relation to iron status. *Eur J Clin Nutr* 2015;69(2):247-55. doi: 10.1038/ejcn.2014.154

**Tan *et al.* Supplementary Material**


18. Grant CC, Wall CR, Brunt D, et al. Population prevalence and risk factors for iron deficiency in Auckland, New Zealand. *J Paediatr Child Health* 2007;43(7-8):532-8. doi: 10.1111/j.1440-1754.2007.01129.x
19. Higgins V, Omidi A, Tahmasebi H, et al. Marked Influence of Adiposity on Laboratory Biomarkers in a Healthy Cohort of Children and Adolescents. *J Clin Endocrinol Metab* 2020;105(4) doi: 10.1210/clinem/dgz161
20. Kassem E, Na'amnih W, Shapira M, et al. Comparison between School-Age Children with and without Obesity in Nutritional and Inflammation Biomarkers. *J Clin Med* 2022;11(23):6973. doi: 10.3390/jcm11236973
21. Manios Y, Moschonis G, Chrousos GP, et al. The double burden of obesity and iron deficiency on children and adolescents in Greece: the Healthy Growth Study. *J Hum Nutr Diet* 2013;26(5):470-8. doi: 10.1111/jhn.12025
22. Moschonis G, Chrousos GP, Lionis C, et al. Association of total body and visceral fat mass with iron deficiency in preadolescents: the Healthy Growth Study. *Br J Nutr* 2012;108(4):710-9. doi: 10.1017/S0007114511005952
23. Nead KG, Halterman JS, Kaczorowski JM, et al. Overweight children and adolescents: a risk group for iron deficiency. *Pediatrics* 2004;114(1):104-8. doi: 10.1542/peds.114.1.104
24. Ortiz Perez M, Vazquez Lopez MA, Ibanez Alcalde M, et al. Relationship between Obesity and Iron Deficiency in Healthy Adolescents. *Child Obes* 2020;16(6):440-47. doi: 10.1089/chi.2019.0276
25. Pompano LM, Correa-Burrows P, Burrows R, et al. Adjusting Ferritin Concentrations for Nonclinical Inflammation in Adolescents with Overweight or Obesity. *J Pediatr* 2022;244:125-32.e1. doi: 10.1016/j.jpeds.2022.01.012
26. Shattnawi KK, Alomari MA, Al-Sheyab N, Bani Salameh A. The relationship between plasma ferritin levels and body mass index among adolescents. *Sci Rep* 2018;8(1):15307. doi: 10.1038/s41598-018-33534-4
27. Suteerojntrakool O, Khongcharoensombat T, Chomtho S, et al. Anthropometric Markers and Iron Status of 6-12-Year-Old Thai Children: Associations and Predictors. *J Nutr Metab* 2021;2021 doi: 10.1155/2021/9629718
28. Sypes EE, Parkin PC, Birken CS, et al. Higher Body Mass Index Is Associated with Iron Deficiency in Children 1 to 3 Years of Age. *J Pediatr* 2019;207:198-204 e1. doi: 10.1016/j.jpeds.2018.11.035
29. Thillan K, Lanerolle P, Thoradeniya T, et al. Micronutrient status and associated factors of adiposity in primary school children with normal and high body fat in Colombo municipal area, Sri Lanka. *BMC Pediatrics* 2021;21(1):14. doi: 10.1186/s12887-020-02473-3
30. Tussing-Humphreys LM, Liang H, Nemeth E, et al. Excess adiposity, inflammation, and iron-deficiency in female adolescents. *J Am Diet Assoc* 2009;109(2):297-302. doi: 10.1016/j.jada.2008.10.044
31. Yalcin SS, Firat MC, Tosun E, Yalcin S. A possible etiological factor in obesity: element status in blood and tooth of overweight versus normal-weight children. *Int J Environ Health Res* 2018:1-13. doi: 10.1080/09603123.2018.1531115
32. Alaofe H, Burney J, Naylor R, Taren D. Prevalence of anaemia, deficiencies of iron and vitamin A and their determinants in rural women and young children: a cross-sectional study in Kalale district of northern Benin. *Public Health Nutr* 2017;20(7):1203-13. doi: 10.1017/S1368980016003608
33. Al-Hussaini AA, Alshehry Z, AlDehaimi A, Bashir MS. Vitamin D and iron deficiencies among Saudi children and adolescents: A persistent problem in the 21st century. *Saudi Journal of Gastroenterology* 2022;28(2):157-64. doi: 10.4103/sjg.sjg_298_21
34. AndrÉ HP, Vieira SA, Franceschini SdCC, et al. Factors associated with the iron nutritional status of Brazilian children aged 4 to 7 years. *Revista de Nutrição* 2017;30(3):345-55. doi: 10.1590/1678-98652017000300007

**Tan *et al.* Supplementary Material**


35. Chitekwe S, Parajuli KR, Paudyal N, et al. Individual, household and national factors associated with iron, vitamin A and zinc deficiencies among children aged 6–59 months in Nepal. *Matern Child Nutr* 2022;18(S1) doi: 10.1111/mcn.13305
36. Ernawati F, Syauqy A, Arifin AY, et al. Micronutrient deficiencies and stunting were associated with socioeconomic status in indonesian children aged 6–59 months. *Nutrients* 2021;13(6) doi: 10.3390/nu13061802
37. Ghosh A, Chowdhury SD, Ghosh T. Undernutrition in Nepalese children: a biochemical and haematological study. *Acta Paediatr* 2012;101(6):671-6. doi: 10.1111/j.1651-2227.2012.02613.x
38. Habib MA, Black K, Soofi SB, et al. Prevalence and Predictors of Iron Deficiency Anemia in Children under Five Years of Age in Pakistan, A Secondary Analysis of National Nutrition Survey Data 2011-2012. *PLoS One* 2016;11(5):e0155051. doi: 10.1371/journal.pone.0155051
39. Kurniawan YA, Muslimatun S, Achadi EL, Sastroamidjojo S. Anaemia and iron deficiency anaemia among young adolescent girls from the peri urban coastal area of Indonesia. *Asia Pac J Clin Nutr* 2006;15(3):350-6.
40. Khatib IM, Elmadfa I. High prevalence rates of anemia, vitamin A deficiency and stunting imperil the health status of Bedouin schoolchildren in North Badia, Jordan. *Ann Nutr Metab* 2009;55(4):358-67. doi: 10.1159/000258632
41. Kumari N, Goyal M, Tiwari RK. Correlation of Serum Biochemical Parameters and Oxidative Stress in Malnourished Children: A Case-control Study. *Journal of Clinical and Diagnostic Research* 2022;16(10):BC01-BC05. doi: 10.7860/JCDR/2022/58226.16890
42. Matsungo TM, Kruger HS, Faber M, et al. The prevalence and factors associated with stunting among infants aged 6 months in a peri-urban South African community. *Public Health Nutr* 2017;20(17):3209-18. doi: 10.1017/S1368980017002087
43. Orsango AZ, Habtu W, Lejisa T, et al. Iron deficiency anemia among children aged 2–5 years in southern Ethiopia: a community-based cross-sectional study. *PeerJ* 2021 doi: 10.7717/peerj.11649
44. Park JS, Chang JY, Hong J, et al. Nutritional zinc status in weaning infants: association with iron deficiency, age, and growth profile. *Biol Trace Elem Res* 2012;150(1-3):91-102. doi: 10.1007/s12011-012-9509-3
45. Sethy PGS, Bulliyya G, Rautray TR, et al. Nutritional Status of Preschool Children in Association with Some Trace Elements in Rural Gram Panchayatas of Bhubaneswar, Odisha, India. *Adv Sci Lett* 2014;20(3):868-73. doi: 10.1166/asl.2014.5414
46. Shukla P, Pandey SK, Singh J, et al. Clinico-Etiopathogenesis of Vitamin B12, Folic Acid and Iron Deficiency in Severe Acute Malnutrition Children: A Tertiary Care Hospital Experience from Central India. *Indian J Clin Biochem* 2023 doi: 10.1007/s12291-022-01100-5
47. Dehghani SM, Katibeh P, Haghighat M, et al. Prevalence of zinc deficiency in 3-18 years old children in shiraz-iran. *Iran Red Crescent Med J* 2011;13(1):4-8.
48. Habib A, Molayemat M, Habib A, et al. Vitamin D and Zinc are Interlinked But Affected by Different Growth Factors in Iranian Children and Adolescents: Vitamin D and Zinc in Iranian Children and Adolescents. *Iran J Pediatr* 2022;32(6) doi: 10.5812/ijp-127158
49. Lu J, Zhang H, Cao W, et al. Study on the Zinc Nutritional Status and Risk Factors of Chinese 6-18-Year-Old Children. *Nutrients* 2023;15(7) doi: 10.3390/nu15071685
50. Fan Y, Zhang C, Bu J. Relationship between Selected Serum Metallic Elements and Obesity in Children and Adolescent in the U.S. *Nutrients* 2017;9(2) doi: 10.3390/nu9020104
51. Ho M, Baur LA, Cowell CT, et al. Zinc status, dietary zinc intake and metabolic risk in Australian children and adolescents; Nepean Longitudinal Study. *Eur J Nutr* 2017;56(7):2407-14. doi: 10.1007/s00394-016-1280-3
52. Perrone L, Gialanella G, Moro R, et al. Zinc, copper, and iron in obese children and adolescents. *Nutr Res* 1998;18(2):183-89.

**Tan *et al.* Supplementary Material**


53. Sharif Y, Sadeghi O, Dorosty A, et al. Serum Levels of Vitamin D, Retinol and Zinc in Relation to overweight among Toddlers: Findings from a National Study in Iran. *Arch Iran Med* 2019;22(4):174-81.
54. Zhu Q, Dai Y, Zhang J, et al. Association between serum zinc concentrations and metabolic risk factors among Chinese children and adolescents. *Br J Nutr* 2021;126(10):1529-36. doi: 10.1017/S0007114521000258
55. Zou Y, Zhang R, Huang L, et al. Serum levels of vitamin D, retinol, zinc, and CRP in relation to obesity among children and adolescents. *Eur J Med Res* 2022;27(1):51. doi: 10.1186/s40001-022-00670-7
56. Engle-Stone R, Ndjebayi AO, Nankap M, et al. Stunting prevalence, plasma zinc concentrations, and dietary zinc intakes in a nationally representative sample suggest a high risk of zinc deficiency among women and young children in Cameroon. *J Nutr* 2014;144(3):382-91. doi: 10.3945/jn.113.188383
57. Galetti V, Mitchikpè CE, Kujinga P, et al. Rural Beninese Children Are at Risk of Zinc Deficiency According to Stunting Prevalence and Plasma Zinc Concentration but Not Dietary Zinc Intakes. *J Nutr* 2016;146(1):114-23. doi: 10.3945/jn.115.216606
58. Gibson RS, Manger MS, Krittaphol W, et al. Does zinc deficiency play a role in stunting among primary school children in NE Thailand? *Br J Nutr* 2007;97(1):167-75. doi: 10.1017/S0007114507250445
59. Goyena EA, Maniego MLV, Ducay AJD, et al. Dietary zinc intake and the underlying factors of serum zinc deficiency among preschool children in the Philippines. *Philipp J Sci* 2021;150(3):799-812.
60. Kongsbak K, Wahed MA, Friis H, Thilsted SH. Acute Phase Protein Levels, T. trichiura, and Maternal Education Are Predictors of Serum Zinc in a Cross-Sectional Study in Bangladeshi Children. *J Nutr* 2006;136(8):2262-68. doi: 10.1093/jn/136.8.2262
61. Marasinghe E, Chackrewarthy S, Abeysena C, Rajindrajith S. Micronutrient status and its relationship with nutritional status in preschool children in urban Sri Lanka. *Asia Pac J Clin Nutr* 2015;24(1):144-51. doi: 10.6133/apjcn.2015.24.1.17
62. Nasiri-babadi P, Sadeghian M, Sadeghi O, et al. The association of serum levels of zinc and vitamin D with wasting among Iranian pre-school children. *Eat Weight Disord* 2021;26(1):211-18. doi: 10.1007/s40519-019-00834-1
63. Naupal-Forcadilla RT, Barba CV, Talavera MTM, Dy MR. Determinants of Zinc Status of 2-3-Year-Old Children in Laguna, Philippines. *Malays J Nutr* 2017;23(1)
64. Okafor AMA, Ikwumere CM, Egumgbe UD, et al. Prevalence and determining factors of stunting among school-aged children in a rural nigerian community: A cross-sectional study. *Curr Res Nutr Food Sci* 2021;9(2):409-22. doi: 10.12944/CRNFSJ.9.2.05
65. Tessema M, De Groote H, I DB, et al. Soil Zinc Is Associated with Serum Zinc But Not with Linear Growth of Children in Ethiopia. *Nutrients* 2019;11(2) doi: 10.3390/nu11020221
66. Van Nhien N, Yabutani T, Khan NC, et al. Association of low serum selenium with anemia among adolescent girls living in rural Vietnam. *Nutrition* 2009;25(1):6-10. doi: 10.1016/j.nut.2008.06.032
67. Yazbeck N, Hanna-Wakim R, El Rafei R, et al. Dietary Zinc Intake and Plasma Zinc Concentrations in Children with Short Stature and Failure to Thrive. *Ann Nutr Metab* 2016;69(1):9-14. doi: 10.1159/000447648
68. Dallazen C, Tietzmann DC, Da Silva SA, et al. Vitamin A deficiency and associated risk factors in children aged 12-59 months living in poorest municipalities in the South Region of Brazil. *Public Health Nutr* 2023;26(1):132-42. doi: 10.1017/S1368980022000325
69. Disalvo L, Varea A, Matamoros N, et al. Vitamin A deficiency and associated factors in preschoolers from the outskirts of La Plata, Buenos Aires. *Arch Argent Pediatr* 2019;117(1):19-25. doi: 10.5546/aap.2019.eng.19
70. de Souza Valente da Silva L, Valeria da Veiga G, Ramalho RA. Association of serum concentrations of retinol and carotenoids with overweight in children and adolescents. *Nutrition* 2007;23(5):392-7. doi: 10.1016/j.nut.2007.02.009

**Tan *et al.* Supplementary Material**


71. Gunanti IR, Marks GC, Al-Mamun A, Long KZ. Low serum concentrations of carotenoids and vitamin E are associated with high adiposity in Mexican-American children. *J Nutr* 2014;144(4):489-95. doi: 10.3945/jn.113.183137
72. Hu W, Tong S, Oldenburg B, Feng X. Serum vitamin A concentrations and growth in children and adolescents in Gansu Province, China. *Asia Pac J Clin Nutr* 2001;10(1):63-6. doi: 10.1046/j.1440-6047.2001.00208.x
73. Ortega-Senovilla H, de Oya M, Garces C. Relationship of NEFA concentrations to RBP4 and to RBP4/retinol in prepubertal children with and without obesity. *J Clin Lipidol* 2019;13(2):301-07. doi: 10.1016/j.jacl.2019.01.006
74. Paes-Silva RP, Gadelha P, Lemos M, et al. Adiposity, inflammation and fat-soluble vitamins in adolescents. *J Pediatr* 2019;95(5):575-83. doi: 10.1016/j.jped.2018.05.008
75. Tian T, Wang Y, Xie W, et al. Associations between Serum Vitamin A and Metabolic Risk Factors among Eastern Chinese Children and Adolescents. *Nutrients* 2022;14(3):610. doi: 10.3390/nu14030610
76. Wei X, Peng R, Cao J, et al. Serum vitamin A status is associated with obesity and the metabolic syndrome among school-age children in Chongqing, China. *Asia Pac J Clin Nutr* 2016;25(3):563-70. doi: 10.6133/apjcn.092015.03
77. Yang C, Chen J, Liu Z, et al. Association of Vitamin A Status with Overnutrition in Children and Adolescents. *Int J Environ Res Public Health* 2015;12(12):15531-9. doi: 10.3390/ijerph121214998
78. Adamu A, Jiya NM, Ahmed H, et al. Prevalence of Vitamin A Deficiency among Malnourished Children in Usmanu Danfodiyo University Teaching Hospital, Sokoto, Northwestern Nigeria. *Pak J Nutr* 2016;15(9):821-28. doi: 10.3923/pjn.2016.821.828
79. Ahmed F, Rahman A, Noor AN, et al. Anaemia and vitamin A status among adolescent schoolboys in Dhaka City, Bangladesh. *Public Health Nutr* 2006;9(3):345-50. doi: 10.1079/PHN2005858
80. Kurugol Z, Egemen A, Keskinoglu P, et al. Vitamin A deficiency in healthy children aged 6-59 months in Izmir Province of Turkey. *Paediatr Perinat Epidemiol* 2000;14:64-9. doi: 10.1046/j.1365-3016.2000.00229.x
81. Oso OO, Abiodun PO, Omotade OO, Oyewole D. Vitamin A status and nutritional intake of carotenoids of preschool children in Ijaye Orile community in Nigeria. *J Trop Pediatr* 2003;49(1):42-7. doi: 10.1093/tropej/49.1.42
82. Samba C, Tchibindat F, Houze P, et al. Prevalence of infant Vitamin A deficiency and undernutrition in the Republic of Congo. *Acta Trop* 2006;97(3):270-83. doi: 10.1016/j.actatropica.2005.11.008
83. Ssentongo P, Ba DM, Ssentongo AE, et al. Association of vitamin A deficiency with early childhood stunting in Uganda: A population-based cross-sectional study. *PLoS One* 2020;15(5):e0233615. doi: 10.1371/journal.pone.0233615
